# Design of a Sequential Filtering Method Fully Equivalent to the Centralized Filter with Cross-Correlated Noise

**DOI:** 10.3390/s26154815

**Published:** 2026-07-29

**Authors:** Yanpeng Huang, Weichang Huang, Chenglin Wen

**Affiliations:** 1 College of Automation, Guangdong University of Petrochemical Technology, Maoming 525000, China; huang.yp@gdupt.edu.cn (Y.H.); huangwc@gdupt.edu.cn (W.H.); 2 Guangdong Provincial Key Laboratory of Petrochemical Equipment Fault Diagnosis, Guangdong University of Petrochemical Technology, Maoming 525000, China

**Keywords:** multi-sensor system, sequential fusion filter, centralized fusion filter, cross-correlated noises

## Abstract

This paper investigates the optimal fusion estimation problem for a class of multi-sensor systems, in which the measurement noises of distinct sensors are mutually cross-correlated and each measurement noise is correlated with the system process noise at the previous time step. First, based on the Gram–Schmidt orthogonalization principle, the innovation of each arriving measurement is recursively projected onto the span of all previous random innovations, and these projections are subtracted to obtain a mutually orthogonal sequential innovation sequence. Second, a sequential fusion Kalman filter is established using this sequential innovation sequence. It is rigorously proved that the proposed sequential fusion Kalman filter is equivalent to the centralized fusion Kalman filter in estimation performance. Finally, a simulation example of a target tracking system is presented to demonstrate the effectiveness of the proposed algorithm.

## 1. Introduction

With the advancement of technology, complex engineering scenarios impose stringent requirements on the accuracy, reliability, and monitoring range of state estimation. However, a single sensor suffers from inherent limitations, such as limited measurement dimensions, weak anti-interference capability, and restricted applicability. Consequently, it can neither meet the demand for high-precision estimation nor cope with comprehensive monitoring of vast areas or complex targets [1]. Against this background, multi-sensor information fusion technology has emerged and rapidly developed. It finds widespread applications in target detection and tracking, signal processing, inertial navigation [2], human activity recognition [3], unmanned systems [4], and the Internet of Things [5].

In centralized fusion, all raw measurements are sent to a fusion center to produce optimally accurate estimates. However, this method entails high computational cost and strict reliability requirements. To address these limitations, researchers have proposed distributed fusion methods [6,7,8]. Each sensor first calculates local estimates independently and transmits the results to the fusion center for final integration. Depending on the fusion criterion, this approach can achieve globally optimal or suboptimal performance and is widely applied in signal processing, communications, and electronics. Commonly employed distributed fusion methods include matrix-weighted [9], diagonal matrix-weighted [10], and scalar-weighted [11] approaches. Representative algorithms include the variance-constrained federated fusion estimator [12], the best linear unbiased estimation fusion rule [13], and covariance intersection fusion [14].

Most existing centralized and distributed fusion algorithms adopt batch processing, which waits for all data or local estimates to be received before computation, potentially introducing significant latency. When network delays occur, neither batch nor conventional sequential processing is suitable. In contrast, sequential fusion processes measurements incrementally as they arrive, eliminating the need to await full data reception. While achieving accuracy comparable to centralized fusion, sequential fusion reduces computational complexity, enhances processing efficiency, and offers better adaptability to asynchronous fusion and delayed data handling [15].

In standard Kalman filtering, it is ideally assumed that the system noise and the measurement noise are independent [16]. Based on this uncorrelated condition, several approaches have been developed, such as the sequential measurement fusion filter [17], variational Bayesian-based sequential centralized fusion estimation [18], and a hybrid robust sequential fusion method for mobile asynchronous WSNs [19]. All of these methods achieve performance equivalent to the centralized fusion filter under the minimum mean square error criterion. In practical linear multi-sensor systems, noise correlations exhibit complex cross-coupling characteristics: the process noise is not only statistically correlated with the measurement noise of each sensor, but the measurement noises of different sensors are also correlated with each other. In this paper, such a scenario is referred to as a dual-correlated noise system, i.e., a system in which both process–measurement and inter-measurement noise correlations exist simultaneously. Examples include noise coupling caused by evaporation ducts in marine environments [20] and discretization of continuous-time systems [21].

For linear multi-sensor systems where the process noise is uncorrelated with the measurement noise but the measurement noises of different sensors are correlated, Ref. [22] equivalently reconstructs the measurement equation. However, this operation couples the original process noise with the newly reconstructed measurement noise in the state estimation error of the resulting sequential filter. For dual-correlated noise scenarios, the fundamental reason why existing sequential filters fail is that the coexistence of process–measurement correlation and inter-measurement correlation destroys the orthogonality of sequentially generated innovations. Consequently, the methods in Refs. [23,24,25,26] have various limitations. Ref. [23] attempts to solve the fusion problem by augmenting the system state and adopting the Gram–Schmidt principle, but it requires the noise covariance matrix to be positive definite. Ref. [24] proposes a Kalman filter based on sequential prediction fusion, yet its performance remains suboptimal. Ref. [25] presents a sequential filter with an extra cross-correlation correction term to characterize noise correlations, which cannot realize strict orthogonalization of the sequential innovation sequence. Ref. [26] confuses the definitions of deterministic and random innovations, thus breaking the orthogonality of innovations and losing global optimality. Consequently, to the best of our knowledge, a sequential fusion Kalman filter fully equivalent to the centralized Kalman filter has not been developed for linear multi-sensor systems with dual-correlated noises.

Motivated by the above discussions, this paper proposes a sequential fusion Kalman filter that is equivalent to the centralized fusion Kalman filter for linear multi-sensor systems with dual-correlated noises. The main contributions are as follows: (1) The dual-correlated noise problem is recast as a stepwise orthogonalization of innovations using the Gram–Schmidt orthogonalization method. (2) A recursive sequential fusion filter based on innovation orthogonalization is developed, achieving lower computational complexity and reduced memory requirements compared to its centralized counterpart. (3) It is rigorously proved that the proposed sequential fusion Kalman filter attains the same estimation performance as the centralized fusion Kalman filter.

Notations: E{•} denotes mathematical expectation; $\delta_{k,l}$ represents the Kronecker delta function; superscripts T and −1 denote matrix transpose and inverse, respectively; $R^{n}$ represents the *n*-dimensional Euclidean space; diag(•) denotes a diagonal matrix; subscript *i* indicates the *i*th sensor; *N* denotes the number of sensors; $I_{n}$ represents the *n*-dimensional identity matrix.

## 2. Problem Statement

Consider a linear dual-correlated dynamic system consisting of multiple sensors, described as follows:

(1) x(k+1)=Φ(k)x(k)+w(k)

(2) $$y_{i}\left(k\right)=H_{i}\left(k\right)x\left(k\right)+v_{i}\left(k\right),\;\;\;\;i=1,2,\cdots ,N\;$$

where $x\left(k\right)\in R^{n}$ are the state variables of the system, $y_{i}\left(k\right)\in R^{q_{i}}$ is the measurement vector of sensor *i*, $\Phi \left(k\right)\in R^{n\times n}$ is the state transition matrix, and $H_{i}\left(k\right)$ is the measurement matrix of appropriate dimension; $w\left(k\right)\in R^{n}$ and $v_{i}\left(k\right)\in R^{q_{i}}$ denote the process noise of the system state and the measurement noise of sensor *i*, respectively.

For Systems (1) and (2), this paper employs the following two assumptions.

**Assumption 1.**  *The system state noise and measurement noise satisfy the following statistical properties: E{w(k)}=0, $E\{v_{i}\left(k\right)\}=0$, $E\left\{w\left(k\right)w^{T}\left(k\right)\right\}=Q_{w}\left(k\right)\delta_{kl}$, $E\left\{v_{i}\left(k\right)v_{j}^{T}\left(l\right)\right\}=R_{ij}\left(k\right)\delta_{kl}$, $E\left\{w\left(l\right)v_{j}{\left(k\right)}^{T}\right\}=S_{i}\left(k\right)\delta_{l,k-1}$.*

**Assumption 2.**  *The initial state x(0) is uncorrelated with w(k) and $v_{i}\left(k\right)$, and it satisfies*

$$E\left\{x\left(0\right)\right\}=\mu_{0},\;E\left\{\left[x\left(0\right)-\mu_{0}\right]{\left[x\left(0\right)-\mu_{0}\right]}^{T}\right\}=P_{0}$$

## 3. Centralized Kalman Filter Fusion for State Estimation in Multi-Sensor Systems with Correlated Noises

This section presents the centralized fusion filtering algorithm for multi-sensor systems with correlated noises. First, the multi-sensor system governed by the linear dynamic Equations (1) and (2) is reformulated into an integrated measurement system.

(3) $$y^{\left(c\right)}\left(k\right)=H^{\left(c\right)}\left(k\right)x\left(k\right)+v^{\left(c\right)}\left(k\right)$$

where

$$y^{\left(c\right)}\left(k\right)={\left[\begin{array}{cccc} y_{1}^{T}\left(k\right) & y_{2}^{T}\left(k\right) & \cdots & y_{N}^{T}\left(k\right) \end{array}\right]}^{T}$$

$$H^{\left(c\right)}\left(k\right)={\left[\begin{array}{cccc} H_{1}^{T}\left(k\right) & H_{2}^{T}\left(k\right) & \cdots & H_{N}^{T}\left(k\right) \end{array}\right]}^{T}$$

$$v^{\left(c\right)}\left(k\right)={\left[\begin{array}{cccc} v_{1}^{T}\left(k\right) & v_{2}^{T}\left(k\right) & \cdots & v_{N}^{T}\left(k\right) \end{array}\right]}^{T}$$

The statistical properties of the state noise w(k) and measurement noise $v^{\left(c\right)}\left(k\right)$ are as follows:

$$\;S\left(k\right)=E\left\{w\left(k-1\right)v^{\left(c\right)}{\left(k\right)}^{T}\right\}=\left[S_{1}\left(k\right),\cdots ,S_{l}\left(k\right),\cdots ,S_{N}\left(k\right)\right]$$

$$\;R\left(k\right)=E\left\{v^{\left(c\right)}{v^{\left(c\right)}}^{T}\right\}=\left[\begin{array}{ccccc} R_{11}\left(k\right) & \cdots & R_{1l}\left(k\right) & \cdots & R_{1N}\left(k\right)\\ \vdots & \ddots & \vdots & \ddots & \vdots \\ R_{l1}\left(k\right) & \cdots & R_{ll}\left(k\right) & \cdots & R_{lN}\left(k\right)\\ \vdots & \ddots & \vdots & \ddots & \vdots \\ R_{N1}\left(k\right) & \cdots & R_{Nl}\left(k\right) & \cdots & R_{NN}\left(k\right) \end{array}\right]$$

$$R_{ll}\left(k\right)=R_{l}\left(k\right);l=1,2,\cdots ,N$$

First, based on the statistical characteristics of the initial state value x(0) and the obtained state measurement sequence $y^{\left(c\right)}\left(k-1\right)$, it is assumed that the estimate of the state x(k) and the corresponding covariance matrix have been acquired.

(4) $${\hat{x}}^{\left(c\right)}\left(k-1|k-1\right)=E\left\{x\left(k-1\right)|{\hat{x}}_{0};\;y^{\left(c\right)}\left(k-1\right)\right\}$$

(5) $$P^{\left(c\right)}\left(k-1|k-1\right)=E\left\{\left[x\left(k-1\right)-{\hat{x}}^{\left(c\right)}\left(k-1|k-1\right)\right]\;{\left[x\left(k-1\right)-{\hat{x}}^{\left(c\right)}\left(k-1|k-1\right)\right]}^{T}\right\}$$

(1) Based on the system state models in Equations (1), (4) and (5), we will respectively obtain the state prediction estimate, the state prediction estimation error, and the state prediction error covariance matrix for the state variable.

(6) $${\hat{x}}^{\left(c\right)}\left(k|k-1\right)=\Phi \left(k-1\right)\;{\hat{x}}^{\left(c\right)}\left(k-1|k-1\right)$$

(7) $$\begin{array}{cc} {\tilde{x}}^{\left(c\right)}\left(k|k-1\right) & =x\left(k\right)-{\hat{x}}^{\left(c\right)}\left(k|k-1\right)\\ \; & =\Phi \left(k-1\right)\;{\tilde{x}}^{\left(c\right)}\left(k-1|k-1\right)+w\left(k-1\right) \end{array}$$

(8) $$\begin{array}{cc} P^{\left(c\right)}\left(k|k-1\right) & =E\left\{{\tilde{x}}^{\left(c\right)}\left(k|k-1\right)\;{\tilde{x}}^{(c)T}\left(k|k-1\right)\right\}\\ \; & =\Phi \left(k-1\right)\;P^{\left(c\right)}\left(k-1|k-1\right)\;\Phi^{T}\left(k-1\right)+Q_{w}\left(k-1\right) \end{array}$$

(2) Based on the integrated observation model (3), the measurement prediction estimation error of the measured value $y^{\left(c\right)}\left(k\right)$ is obtained.

(9) $$\begin{array}{cc} \varepsilon^{\left(c\right)}\left(k\right) & =y^{\left(c\right)}\left(k\right)-H^{\left(c\right)}\left(k\right)\;{\hat{x}}^{\left(c\right)}\left(k|k-1\right)\\ \; & =H^{\left(c\right)}\left(k\right)\;{\tilde{x}}^{\left(c\right)}\left(k|k-1\right)+v^{\left(c\right)}\left(k\right) \end{array}$$

(3) Design the centralized fusion Kalman filter for estimating the state variable x(k)

(10) $$\begin{array}{c} {\hat{x}}^{\left(c\right)}(k|k)={\hat{x}}^{\left(c\right)}(k|k-1)+K^{\left(c\right)}\left(k\right)\varepsilon^{\left(c\right)}\left(k\right) \end{array}$$

where $K^{\left(c\right)}\left(k\right)$ is the gain matrix of the centralized fusion Kalman filter to be determined.

(4) Solve for the gain matrix of the centralized fusion Kalman filter.

Compute the estimation error vector ${\tilde{x}}^{\left(c\right)}(k|k)$ containing the gain matrix to be determined.

(11) $$\begin{array}{cc} {\tilde{x}}^{\left(c\right)}\left(k|k\right) & =x\left(k\right)-{\hat{x}}^{\left(c\right)}\left(k|k-1\right)\\ \; & ={\tilde{x}}^{\left(c\right)}\left(k|k-1\right)-K^{\left(c\right)}\left(k\right)\;\varepsilon^{\left(c\right)}\left(k\right) \end{array}$$

Using the statistical orthogonality principle between random variables,

(12) $$E\left\{{\tilde{x}}^{\left(c\right)}\left(k|k\right)\;\varepsilon^{\left(c\right)}{\left(k\right)}^{T}\right\}=0$$

The analytical expression of the gain matrix $K^{\left(c\right)}\left(k\right)$ in the filter of Equation (11) is obtained:

(13) $$K^{\left(c\right)}\left(k\right)=\left[P\left(k|k-1\right)\;H^{\left(c\right)}{\left(k\right)}^{T}+S\left(k\right)\right]\;Q_{\varepsilon^{\left(c\right)}}{\left(k\right)}^{-1}$$

where

$$\begin{array}{c} Q_{\varepsilon^{\left(c\right)}}\left(k\right)=E\left\{\varepsilon^{\left(c\right)}\left(k\right)\varepsilon^{\left(c\right)}{\left(k\right)}^{T}\right\}\\ \;\;\;\;\;\;\;\;\;\;\;\;\;=H^{\left(c\right)}\left(k\right)P^{\left(c\right)}\left(k|k-1\right)H^{\left(c\right)}{\left(k\right)}^{T}+H^{\left(c\right)}\left(k\right)S\left(k\right)\\ \;\;\;\;\;\;\;\;\;\;\;\;\;+S^{T}\left(k\right)H^{\left(c\right)}{\left(k\right)}^{T}+R\left(k\right) \end{array}$$

(5) Calculate the estimate error covariance matrix of the state variable x(k):

(14) $$\begin{array}{cc} P^{\left(c\right)}\left(k|k\right) & =E\left\{{\tilde{x}}^{\left(c\right)}\left(k|k\right)\;{\tilde{x}}^{(c)T}\left(k|k\right)\right\}\\ \; & =P^{\left(c\right)}\left(k|k-1\right)-K^{\left(c\right)}\left(k\right)\;Q_{\varepsilon^{\left(c\right)}}\left(k\right)\;K^{\left(c\right)}{\left(k\right)}^{T} \end{array}$$

where the superscript “(c)” in the above formulas denotes the centralized fusion Kalman filter.

The above is the design process of the centralized Kalman fusion filtering method under the condition where the system process noise is correlated with the integrated measurement noise. Considering that, the centralized Kalman fusion filter suffers from some insurmountable issues: (1) high complexity, requiring high-dimensional matrix inversion operations; (2) it is difficult for distributed multiple sensors to arrive simultaneously in real time, and the centralized Kalman fusion filter must wait for delayed measurements to arrive before execution; in particular, when data packet loss occurs, the fusion filter cannot be executed effectively, etc.

To this end, in the following section, we will establish a sequential Kalman fusion filtering method for system state estimation based on measurement data arriving at the fusion center in chronological order. This approach effectively resolves the aforementioned challenges associated with the centralized Kalman fusion filter under the dual-correlated noise condition in linear dynamic systems—where the system process noise exhibits statistical correlation not only with all distributed measurement noises but also among the individual measurement noises across different sensors. To the best of our knowledge, few existing sequential Kalman fusion filters can realize both formal and performance equivalence to the centralized counterpart for linear dynamic systems with dual-correlated noises. This motivates the method proposed in this paper.

## 4. Comparison of Existing Sequential Fusion Methods and Research Motivation

Refs. [22,27] only consider scenarios where measurement noises are cross-correlated among different sensors. Ref. [23] reconstructs the measurement equation via observation augmentation. However, this method requires the noise covariance matrix to be positive definite, which limits its practical applicability. Ref. [28] transforms the state equation combined with the Gram–Schmidt orthogonalization to realize the equivalent form of the measurement equation. Despite different design ideas, the above three approaches all feature high computational complexity and large memory consumption, restricting their practical engineering applications.

Ref. [24] proposes a Kalman filter based on sequential prediction fusion. Ref. [25] introduces additional cross-correlation correction terms to characterize noise coupling and further develops a sequential filter. Nevertheless, it fails to establish an effective innovation decorrelation mechanism for linear systems with dual-correlated noises, resulting in correlated sequential innovations. Both methods only achieve suboptimal estimation performance.

As a representative work in this field, ref. [26] presents a sequential fusion Kalman filter. However, the definition and application of the core concept “innovation” lack rigor, leading to inconsistencies in theoretical derivation. Thus, its claimed performance cannot be strictly validated. This chapter conducts analysis from three perspectives: (1) distinguishing the definitions and application scenarios of deterministic innovation and random innovation; (2) illustrating the influence of conceptual ambiguity on the global optimality of the filter; (3) analyzing the mechanism behind the non-orthogonality and non-equivalence between sequential innovations and centralized innovations, which eventually leads to performance degradation.

### 4.1. Dual Characteristics of Innovation in Classical Linear Optimal Kalman Filters

In the derivation and implementation of the conventional single-sensor linear Kalman filter, innovation takes two distinct mathematical forms with different theoretical functions and practical applications, namely

(15) $$\begin{array}{c} \varepsilon^{\left(f\right)}\left(k\right)=y\left(k\right)-H\left(k\right)\hat{x}\left(k|k-1\right) \end{array}$$

(16) $$\begin{array}{c} \varepsilon^{\left(r\right)}\left(k\right)=H\left(k\right)\tilde{x}\left(k|k-1\right)+v\left(k\right) \end{array}$$

Among them, the superscript “(f)” in Equation (15) indicates that the “innovation” is regarded as a realized innovation; the superscript “(r)” in Equation (16) indicates that the “innovation” is regarded as a random innovation variable. Moreover, we have $\tilde{x}\left(k|k-1\right)∼N\left(0,P\left(k|k-1\right)\right)$, v(k)∼N(0,R(k)).

**Remark 1.**  *(a) The deterministic innovation $\varepsilon^{\left(f\right)}\left(k\right)$ in Equation (15) is the unused residual in a given measurement y(k). It is a deterministic vector used only during filter execution. (b) The stochastic innovation $\varepsilon^{\left(r\right)}\left(k\right)$ in Equation (16) is a linear combination of $\tilde{x}\left(k|k-1\right)$ and v(k). It is used only in theoretical derivations of optimality. (c) The deterministic vector $\varepsilon^{\left(f\right)}\left(k\right)$ and the stochastic vector $\varepsilon^{\left(r\right)}\left(k\right)$ are neither equivalent nor identical; rather, the deterministic vector $\varepsilon^{\left(f\right)}\left(k\right)$ can only be regarded as a sample realization of the stochastic vector $\varepsilon^{\left(r\right)}\left(k\right)$ obtained from a specific sampling instance, i.e., the relationship $\varepsilon^{\left(f\right)}\left(k\right)=E\left\{\varepsilon^{\left(r\right)}\left(k\right)|\varepsilon^{\left(f\right)}\left(k\right)\right\}$ holds. Furthermore, when expressed in the form $\varepsilon^{\left(f\right)}\left(k\right)=\varepsilon^{\left(r\right)}\left(k\right)$, the statistical properties of the two random vectors $E\{\tilde{x}\left(k|k-1\right)\}=0$ and E{v(k)}=0 in Equation (16) are altered.*

**Corollary 1.**  *Under the condition of $\varepsilon^{\left(f\right)}\left(k\right)=\varepsilon^{\left(r\right)}\left(k\right)$, the random variables $\tilde{x}\left(k|k-1\right)$ and v(k) exhibit the following new statistical properties, respectively:*

(17) $$\tilde{x}\left(k|k-1\right)∼N\left[{\hat{\tilde{x}}}_{\tilde{x}|\varepsilon^{\left(f\right)}}\left(k|k-1\right),\;P_{\tilde{x}|\varepsilon^{\left(f\right)}}\left(k|k-1\right)\right]\;v\left(k\right)∼N\left[{\hat{v}}_{v|\varepsilon^{\left(f\right)}}\left(k\right),\;R_{\tilde{x}|\varepsilon^{\left(f\right)}}\left(k\right)\right]$$

*where, based on the least squares method or the traditional Kalman filtering method, their statistical properties can be obtained, respectively.*

(18) $$\begin{array}{cc} {\hat{\tilde{x}}}_{\tilde{x}|\varepsilon^{\left(f\right)}}\left(k|k-1\right) & =E\left\{\tilde{x}\left(k|k-1\right)|\varepsilon^{\left(f\right)}\left(k\right)\right\}\\ \; & =-P\left(k|k-1\right)H{\left(k\right)}^{T}{\left[H\left(k\right)P\left(k|k-1\right)H{\left(k\right)}^{T}+R\left(k\right)\right]}^{-1}\varepsilon^{\left(f\right)}\left(k\right) \end{array}$$

(19) $$\begin{array}{cc} P_{\tilde{x}|\varepsilon^{\left(f\right)}}\left(k|k-1\right) & =E\left\{\left[\tilde{x}\left(k|k-1\right)-{\hat{\tilde{x}}}_{\tilde{x}|\varepsilon^{\left(f\right)}}\left(k|k-1\right)\right]{\left[\tilde{x}\left(k|k-1\right)-{\hat{\tilde{x}}}_{\tilde{x}|\varepsilon^{\left(f\right)}}\left(k|k-1\right)\right]}^{T}\right\}\\ \; & =P\left(k|k-1\right)-P\left(k|k-1\right)H^{T}\left(k\right){\left[H\left(k\right)P\left(k|k-1\right)H^{T}\left(k\right)+R\left(k\right)\right]}^{-1}\\ \; & \;\times H(k)P(k|k-1) \end{array}$$

(20) $$\begin{array}{cc} {\hat{v}}_{v|\varepsilon^{\left(f\right)}}\left(k\right) & =E\left\{v\left(k\right)|\varepsilon^{\left(f\right)}\left(k\right)\right\}\\ \; & =R\left(k\right){\left[H\left(k\right)P\left(k|k-1\right)H{\left(k\right)}^{T}+R\left(k\right)\right]}^{-1}\varepsilon^{\left(f\right)}\left(k\right) \end{array}$$

(21) $$\begin{array}{cc} P_{v|\varepsilon^{\left(f\right)}}\left(k|k-1\right) & =E\left\{\left[v\left(k\right)-{\hat{v}}_{v|\varepsilon^{\left(f\right)}}\left(k\right)\right]{\left[v\left(k\right)-{\hat{v}}_{v|\varepsilon^{\left(f\right)}}\left(k\right)\right]}^{T}\right\}\\ \; & =R\left(k\right)-R\left(k\right){\left[H\left(k\right)P\left(k|k-1\right)H^{T}\left(k\right)+R\left(k\right)\right]}^{-1}R\left(k\right)\\ \; & =H\left(k\right)P_{\tilde{x}|\varepsilon^{\left(f\right)}}\left(k|k-1\right)H^{T}\left(k\right) \end{array}$$

### 4.2. Theoretical Characteristics and Limitations of Typical Existing Methods

#### 4.2.1. Usage Limitations of the Innovation Concept in Ref. [26]

The distinction between realized innovation and random innovation variable is insufficient.

(a) In Equation (13) of Theorem 1 in Ref. [26], the realized innovation for Sensor 1 and Sensor 2 is given, which is mainly used during the execution of the filter.

(22) $$\varepsilon_{1}^{\left(s,f\right)}\left(k\right)=\varepsilon_{1}\left(k\right)=y_{1}\left(k\right)-H_{1}\left(k\right)\;\hat{x}\left(k|k-1\right)$$

(23) $$\varepsilon_{2}^{\left(s,f\right)}\left(k\right)=\varepsilon_{2}\left(k\right)=y_{2}\left(k\right)-H_{2}\left(k\right)\;{\hat{x}}_{1}\left(k\right)-{\hat{v}}_{2|1}\left(k\right)$$

where the expression ${\hat{v}}_{2|1}\left(k\right)$ in Equation (23) corresponds to Equation (16) in Ref. [26], i.e.,

$${\hat{v}}_{2|1}\left(k\right)=M_{2|1}\left(k\right)\varepsilon_{1}^{\left(s,f\right)}\left(k\right)=M_{2|1}\left(k\right)E\left\{\varepsilon_{1}^{\left(s,r\right)}\left(k\right)|\varepsilon_{1}^{\left(s,f\right)}\left(k\right)\right\}$$

As explained in Remark 1, $\varepsilon_{1}^{\left(s,f\right)}\left(k\right)$ and $\varepsilon_{2}^{\left(s,f\right)}\left(k\right)$ are also used only during the execution of the filter. (b) In Ref. [26], the random innovation variable is also provided, which is primarily used in the derivation and establishment process of the filter.

(24) $$\varepsilon_{1}^{\left(s,r\right)}\left(k\right)=H_{1}\left(k\right)\;\tilde{x}\left(k|k-1\right)+v_{1}\left(k\right)$$

(25) $$\varepsilon_{2}^{\left(s,r\right)}\left(k\right)=H_{2}\left(k\right)\;{\tilde{x}}_{1}\left(k\right)+{\tilde{v}}_{2|1}\left(k\right)$$

The above Equation (25) corresponds to Equation (27) in Ref. [26], i.e.,

$$\varepsilon_{2}\left(k\right)=H_{2}\left(k\right){\tilde{x}}_{1}\left(k\right)+{\tilde{v}}_{2|1}\left(k\right)$$

**Theoretical Analysis** **1.** *We can observe that in Equation (25) ${\tilde{v}}_{2|1}\left(k\right)=v_{2}\left(k\right)-{\hat{v}}_{2|1}\left(k\right)$, where the term “${\hat{v}}_{2|1}\left(k\right)$” should not be ${\hat{v}}_{2|1}\left(k\right)=M_{2|1}\left(k\right)\;\varepsilon_{1}^{\left(s,f\right)}\left(k\right)$, as defined in Equation (23), but should instead be $M_{2|1}\left(k\right)\;\varepsilon_{1}^{\left(s,r\right)}\left(k\right)$, i.e.,*

(26) $${\tilde{v}}_{2|1}\left(k\right)=v_{2}\left(k\right)-M_{2|1}\left(k\right)\;\varepsilon_{1}^{\left(s,r\right)}\left(k\right)$$

*The lack of rigorous distinction between the concepts of innovation led the authors of Ref. [26] to equate $\varepsilon_{1}^{\left(s,f\right)}\left(k\right)$ with $\varepsilon_{1}^{\left(s,r\right)}\left(k\right)$, i.e., $\varepsilon_{1}^{\left(s,f\right)}\left(k\right)=\varepsilon_{1}^{\left(s,r\right)}\left(k\right)$. Similarly, they also regarded $\varepsilon_{2}^{\left(s,f\right)}\left(k\right)$ as identical to $\varepsilon_{2}^{\left(s,r\right)}\left(k\right)$, i.e., $\varepsilon_{2}^{\left(s,f\right)}\left(k\right)=\varepsilon_{2}^{\left(s,r\right)}\left(k\right)$. This understanding consequently leads to the result of Corollary 1.*

(1) Regarding $\varepsilon_{1}^{\left(s,f\right)}\left(k\right)$ and $\varepsilon_{1}^{\left(s,r\right)}\left(k\right)$ as equivalent, and based on Corollary 1, the statistical properties of the random variables $\tilde{x}\left(k|k-1\right)$ and $v_{1}\left(k\right)$ in Equation (25) are derived under the condition $\varepsilon_{1}^{\left(s,f\right)}\left(k\right)$:

(27) $$\tilde{x}\left(k|k-1\right)∼N\left[{\hat{\tilde{x}}}_{\tilde{x}|\varepsilon_{1}^{\left(s,f\right)}}\left(k|k-1\right),\;P_{\tilde{x}|\varepsilon_{1}^{\left(s,f\right)}}\left(k|k-1\right)\right]\;v_{1}\left(k\right)∼N\left[{\hat{v}}_{v_{1}|\varepsilon_{1}^{\left(s,f\right)}}\left(k\right),\;R_{v_{1}|\varepsilon_{1}^{\left(s,f\right)}}\left(k\right)\right]$$

(28) $${\hat{\varepsilon }}_{1}^{\left(s,r\right)}\left(k\right)=E\left\{\varepsilon_{1}^{\left(s,r\right)}\left(k\right)|\varepsilon_{1}^{\left(s,f\right)}\left(k\right)\right\}=H_{1}\left(k\right)\;{\hat{\tilde{x}}}_{\tilde{x}|\varepsilon_{1}^{\left(s,f\right)}}\left(k|k-1\right)+{\hat{v}}_{v_{1}|\varepsilon_{1}^{\left(s,f\right)}}\left(k\right)$$

(2) Regarding $\varepsilon_{2}^{\left(s,f\right)}\left(k\right)$ and $\varepsilon_{2}^{\left(s,r\right)}\left(k\right)$ as equivalent, and based on Corollary 1, the statistical properties of the random variables $\tilde{x}\left(k|k-1\right)$ and $v_{1}\left(k\right)$ in Equation (25) are derived under the condition $\varepsilon_{2}^{\left(s,f\right)}\left(k\right)$:

(29) $${\tilde{x}}_{1}\left(k\right)∼N\left[{\hat{\tilde{x}}}_{{\tilde{x}}_{1}|\varepsilon_{2}^{\left(s,f\right)}}\left(k\right),\;P_{{\tilde{x}}_{1}|\varepsilon_{2}^{\left(s,f\right)}}\left(k|k\right)\right]\;{\tilde{v}}_{2|1}\left(k\right)∼N\left[{\hat{\tilde{v}}}_{{\tilde{v}}_{2|1}|\varepsilon_{2}^{\left(s,f\right)}}\left(k\right),\;R_{{\tilde{v}}_{2|1}|\varepsilon_{2}^{\left(s,f\right)}}\left(k\right)\right]$$

(30) $${\hat{\varepsilon }}_{2}^{\left(s,r\right)}\left(k\right)=E\left\{\varepsilon_{2}^{\left(s,r\right)}\left(k\right)|\varepsilon_{2}^{\left(s,f\right)}\left(k\right)\right\}=H_{2}\left(k\right)\;{\hat{\tilde{x}}}_{{\tilde{x}}_{1}|\varepsilon_{2}^{\left(s,f\right)}}\left(k\right)+{\hat{\tilde{v}}}_{{\tilde{v}}_{2|1}|\varepsilon_{2}^{\left(s,f\right)}}\left(k\right)$$

**Theoretical Analysis** **2.** *Under the definition provided in Equation (26), ${\tilde{v}}_{2|1}\left(k\right)=v_{2}\left(k\right)-{\hat{v}}_{2|1}\left(k\right)=v_{2}\left(k\right)-M_{2|1}\left(k\right)\;\varepsilon_{1}^{\left(s,f\right)}\left(k\right)$, the intermediate random variable ${\tilde{v}}_{2|1}\left(k\right)$ should possess the following statistical properties: ${\tilde{v}}_{2|1}\left(k\right)∼N\left[{\hat{\tilde{v}}}_{2|1}\left(k\right),\;R_{{\tilde{v}}_{2|1}}\left(k\right)\right]$.*

*Proof is given as follows: according to the statistical property of the modeling error $v_{2}\left(k\right)$ corresponding to sensor 2, i.e., $v_{2}\left(k\right)∼N\left[0,R_{2}\left(k\right)\right]$, we have*

(31) $$0=E\left\{v_{2}\left(k\right)\right\}=E\left\{{\hat{v}}_{2|1}\left(k\right)+{\tilde{v}}_{2|1}\left(k\right)\right\}={\hat{v}}_{2|1}\left(k\right)+E\left\{{\tilde{v}}_{2|1}\left(k\right)\right\}$$

(32) $${\hat{\tilde{v}}}_{2|1}\left(k\right)=E\left\{{\tilde{v}}_{2|1}\left(k\right)\right\}=-{\hat{v}}_{2|1}\left(k\right)$$

*Under the assumption ${\tilde{v}}_{2|1}\left(k\right)={\hat{\tilde{v}}}_{2|1}\left(k\right)+{\tilde{\tilde{v}}}_{2|1}\left(k\right)$, we can further derive the properties of the intermediate noise variable ${\tilde{\tilde{v}}}_{2|1}\left(k\right)$:*

(33) $${\tilde{\tilde{v}}}_{2|1}\left(k\right)={\tilde{v}}_{2|1}\left(k\right)-{\hat{\tilde{v}}}_{2|1}\left(k\right)={\tilde{v}}_{2|1}\left(k\right)+{\hat{v}}_{2|1}\left(k\right)\equiv v_{2}\left(k\right)$$

(34) $${\tilde{v}}_{2|1}\left(k\right)={\hat{\tilde{v}}}_{2|1}\left(k\right)+{\tilde{\tilde{v}}}_{2|1}\left(k\right)=-{\hat{v}}_{2|1}\left(k\right)+{\tilde{\tilde{v}}}_{2|1}\left(k\right)=-{\hat{v}}_{2|1}\left(k\right)+v\left(k\right)$$

(35) $$R_{{\tilde{v}}_{2|1}}\left(k\right)=E\left\{{\tilde{v}}_{2|1}\left(k\right)\;{\tilde{v}}_{2|1}{\left(k\right)}^{T}\right\}={\hat{v}}_{2|1}\left(k\right)\;{\hat{v}}_{2|1}{\left(k\right)}^{T}+R_{2}\left(k\right)$$

#### 4.2.2. Analysis of the Orthogonality and Equivalence Relationship of the Innovation in Ref. [26]

**Corollary 2.**  *Owing to Theoretical Analysis 1, the random innovation variables $\varepsilon_{1}\left(k\right)$ and $\varepsilon_{2}\left(k\right)$ are not orthogonal, i.e., $E\{\varepsilon_{1}\left(k\right)\varepsilon_{2}{\left(k\right)}^{T}\}\neq 0$, and proof is given as follows.*

(36) $$\begin{array}{cc} E\{\varepsilon_{1}^{\left(s,r\right)}\left(k\right)\varepsilon_{2}^{\left(s,r\right)}{\left(k\right)}^{T}\}\; & =E\{\varepsilon_{1}^{\left(s,r\right)}\left(k\right){\left[H_{2}\left(k\right){\tilde{x}}_{1}\left(k\right)+v_{2}\left(k\right)-{\hat{v}}_{2|1}\left(k\right)\right]}^{T}\}\\ \; & =E\{\varepsilon_{1}^{\left(s,r\right)}\left(k\right){\left[H_{2}\left(k\right){\tilde{x}}_{1}\left(k\right)+v_{2}\left(k\right)-M_{2|1}\left(k\right)\varepsilon_{1}^{\left(s,f\right)}\left(k\right)\right]}^{T}\}\\ \; & =E\left\{\varepsilon_{1}^{\left(s,r\right)}\left(k\right)v_{2}{\left(k\right)}^{T}\right\}-E\left\{\varepsilon_{1}^{\left(s,r\right)}\left(k\right)\varepsilon_{1}^{\left(s,f\right)}{\left(k\right)}^{T}\right\}M_{2|1}{\left(k\right)}^{T}\\ \; & =E\left\{\left[H_{1}\left(k\right)\tilde{x}\left(k|k-1\right)+v_{1}\left(k\right)\right]v_{2}{\left(k\right)}^{T}\right\}-E\left\{\varepsilon_{1}^{\left(s,r\right)}\left(k\right)\right\}\varepsilon_{1}^{\left(s,f\right)}{\left(k\right)}^{T}M_{2|1}{\left(k\right)}^{T}\\ \; & =H_{1}\left(k\right)S_{2}\left(k\right)+R_{12}\left(k\right)-{\hat{\varepsilon }}_{1}^{\left(s,r\right)}\left(k\right)\varepsilon_{1}^{\left(s,f\right)}{\left(k\right)}^{T}M_{2|1}{\left(k\right)}^{T}\neq 0 \end{array}$$

**Corollary 3.**  *Theoretical Analysis 2 leads to the consequence that an equivalence relationship between the centralized innovation and the sequential innovation cannot be established; that is, there exists no invertible matrix M(k) such that the following equation holds:*

(37) $${\left[\varepsilon_{1}^{\left(c,r\right)}{\left(k\right)}^{T},\;\varepsilon_{2}^{\left(c,r\right)}{\left(k\right)}^{T}\right]}^{T}\neq M\left(k\right){\left[\varepsilon_{1}^{\left(s,r\right)}{\left(k\right)}^{T},\;\varepsilon_{2}^{\left(s,r\right)}{\left(k\right)}^{T}\right]}^{T},\;\left|M\left(k\right)\right|\neq 0$$

**Proof.**  For the two-sensor case, let $v_{2}\left(k\right)={\hat{v}}_{2|1}\left(k\right)+{\tilde{v}}_{2|1}\left(k\right)$. If $v_{2}\left(k\right)$ is removed from the random innovation variable $\varepsilon_{2}^{\left(s,r\right)}\left(k\right)$, then the random innovation variable sequence of the centralized fusion filter, defined as ${\left[B_{1}{\left(k\right)}^{T},B_{2}{\left(k\right)}^{T}\right]}^{T}\equiv {\left[\varepsilon_{1}^{\left(c,r\right)}{\left(k\right)}^{T},\varepsilon_{2}^{\left(c,r\right)}{\left(k\right)}^{T}\right]}^{T}$, will lose its connection with the sequence ${\left[\varepsilon_{1}^{\left(r\right)}{\left(k\right)}^{T},\varepsilon_{2}^{\left(r\right)}{\left(k\right)}^{T}\right]}^{T}$ used in the sequential fusion filter of Ref. [26]. That is, there does not exist an invertible matrix M(k) such that the following equation holds: where the expression $B_{l}\left(k\right),l=1,2$ denotes the random innovation variable designed for sensors 1 and 2, respectively, as defined in Ref. [26] for the centralized Kalman filter under the two-sensor scenario, $\varepsilon_{l}^{\left(c,r\right)}\left(k\right)\equiv B_{l}\left(k\right)$; l=1,2 here for notational consistency. Meanwhile, it holds that $\varepsilon_{1}^{\left(c,r\right)}\left(k\right)\equiv \varepsilon_{1}^{\left(s,r\right)}\left(k\right)$.

Equation (31) in Ref. [26] holds only under the premise that ${\hat{v}}_{2|1}\left(k\right)⊥{\tilde{v}}_{2|1}\left(k\right)$ and ${\tilde{x}}_{1}\left(k\right)⊥{\hat{v}}_{2|1}\left(k\right)$. However, the following derivations show that these two conditions cannot be satisfied. □

**Corollary 4.**  *${\tilde{x}}_{1}\left(k\right)⊥{\hat{v}}_{2|1}\left(k\right)$ do not hold, i.e., $E\{{\tilde{x}}_{1}\left(k\right){\hat{v}}_{2|1}{\left(k\right)}^{T}\}\neq 0$.*

(38) $$\begin{array}{cc} \; & E\{{\tilde{x}}_{1}\left(k\right){\hat{v}}_{2|1}{\left(k\right)}^{T}|\varepsilon_{1}^{\left(s,f\right)}\left(k\right)\}\\ \; & =E\left\{{\tilde{x}}_{1}\left(k\right){\left[M_{2|1}\left(k\right)\varepsilon_{1}^{\left(s,f\right)}\left(k\right)\right]}^{T}|\varepsilon_{1}^{\left(s,f\right)}\left(k\right)\right\}\\ \; & =E\left\{{\tilde{x}}_{1}\left(k\right)|\varepsilon_{1}^{\left(s,f\right)}\left(k\right)\right\}\varepsilon_{1}^{\left(s,f\right)}{\left(k\right)}^{T}M_{2|1}{\left(k\right)}^{T}\\ \; & =\left\{\left[I-K_{1}^{\left(s\right)}\left(k\right)H_{1}\left(k\right)\right]\hat{\tilde{x}}\left(k|k-1\right)+K_{1}^{\left(s\right)}\left(k\right){\hat{v}}_{1}\left(k\right)\right\}{\hat{v}}_{2|1}{\left(k\right)}^{T}\\ \; & =\left[I-K_{1}^{\left(s\right)}\left(k\right)H_{1}\left(k\right)\right]\hat{\tilde{x}}\left(k|k-1\right){\hat{v}}_{2|1}{\left(k\right)}^{T}+K_{1}^{\left(s\right)}\left(k\right){\hat{v}}_{1}\left(k\right){\hat{v}}_{2|1}{\left(k\right)}^{T}\\ \; & \neq 0 \end{array}$$

**Corollary 5.**  *${\hat{v}}_{2|1}\left(k\right)⊥{\tilde{v}}_{2|1}\left(k\right)$ do not hold, i.e., $E\{{\hat{v}}_{2|1}\left(k\right){\tilde{v}}_{2|1}{\left(k\right)}^{T}\}\neq 0$.*

(39) $$\begin{array}{cc} E\{{\hat{v}}_{2|1}\left(k\right){\tilde{v}}_{2|1}{\left(k\right)}^{T}\}\; & =E\left\{{\hat{v}}_{2|1}\left(k\right){\left[{\hat{\tilde{v}}}_{2|1}\left(k\right)+{\tilde{\tilde{v}}}_{2|1}\left(k\right)\right]}^{T}\right\}\\ \; & =E\left\{{\hat{v}}_{2|1}\left(k\right){\hat{\tilde{v}}}_{2|1}{\left(k\right)}^{T}\right\}+E\left\{{\hat{v}}_{2|1}\left(k\right){\tilde{\tilde{v}}}_{2|1}{\left(k\right)}^{T}\right\}\\ \; & ={\hat{v}}_{2|1}\left(k\right){\hat{\tilde{v}}}_{2|1}{\left(k\right)}^{T}+{\hat{v}}_{2|1}\left(k\right)E\left\{{\tilde{\tilde{v}}}_{2|1}{\left(k\right)}^{T}\right\}\\ \; & =-{\hat{v}}_{2|1}\left(k\right){\hat{v}}_{2|1}{\left(k\right)}^{T}\neq 0 \end{array}$$

**Corollary 6.**  *$E\left\{{\tilde{x}}_{1}\left(k\right)v_{2}{\left(k\right)}^{T}\right\}\neq E\left\{{\tilde{x}}_{1}\left(k\right){\tilde{v}}_{2|1}{\left(k\right)}^{T}\right\}$.*

*From Corollary 3,*

(40) $$\begin{array}{cc} \; & E\{{\tilde{x}}_{1}\left(k\right)v_{2}{\left(k\right)}^{T}|\varepsilon_{1}^{\left(s,f\right)}\left(k\right)\}\\ \; & =E\left\{{\tilde{x}}_{1}\left(k\right){\left[{\hat{v}}_{2|1}\left(k\right)+{\tilde{v}}_{2|1}\left(k\right)\right]}^{T}|\varepsilon_{1}^{\left(s,f\right)}\left(k\right)\right\}\\ \; & =E\left\{{\tilde{x}}_{1}\left(k\right){\hat{v}}_{2|1}\left(k\right)|\varepsilon_{1}^{\left(s,f\right)}\left(k\right)\right\}+E\left\{{\tilde{x}}_{1}\left(k\right){\tilde{v}}_{2|1}{\left(k\right)}^{T}|\varepsilon_{1}^{\left(s,f\right)}\left(k\right)\right\}\\ \; & =E\left\{{\tilde{x}}_{1}\left(k\right)|\varepsilon_{1}^{\left(s,f\right)}\left(k\right)\right\}{\hat{v}}_{2|1}{\left(k\right)}^{T}+E\left\{{\tilde{x}}_{1}\left(k\right){\tilde{v}}_{2|1}{\left(k\right)}^{T}|\varepsilon_{1}^{\left(s,f\right)}\left(k\right)\right\}\\ \; & ={\hat{\tilde{x}}}_{{\tilde{x}}_{1}|\varepsilon_{1}^{\left(s,f\right)}}\left(k\right){\hat{v}}_{2|1}{\left(k\right)}^{T}+E\left\{{\tilde{x}}_{1}\left(k\right){\tilde{v}}_{2|1}{\left(k\right)}^{T}|\varepsilon_{1}^{\left(s,f\right)}\left(k\right)\right\}\\ \; & \neq E\{{\tilde{x}}_{1}\left(k\right){\tilde{v}}_{2|1}{\left(k\right)}^{T}|\varepsilon_{1}^{\left(s,f\right)}\left(k\right)\} \end{array}$$

**Corollary 7.** 
$E\left\{v_{2}\left(k\right){\tilde{v}}_{2|1}{\left(k\right)}^{T}\right\}\neq E\left\{{\tilde{v}}_{2|1}\left(k\right){\tilde{v}}_{2|1}{\left(k\right)}^{T}\right\}$

*From Corollary 3, $v_{2}\left(k\right)={\hat{v}}_{2|1}\left(k\right)+{\tilde{v}}_{2|1}\left(k\right)$, ${\hat{\tilde{v}}}_{2|1}\left(k\right)=-{\hat{v}}_{2|1}\left(k\right)$,*

(41) $$\begin{array}{cc} \; & E\{v_{2}\left(k\right){\tilde{v}}_{2|1}{\left(k\right)}^{T}\}\\ \; & =E\{\left[{\hat{v}}_{2|1}\left(k\right)+{\tilde{v}}_{2|1}\left(k\right)\right]{\tilde{v}}_{2|1}{\left(k\right)}^{T}\}\\ \; & =E\left\{{\hat{v}}_{2|1}\left(k\right){\tilde{v}}_{2|1}{\left(k\right)}^{T}\right\}+E\left\{{\tilde{v}}_{2|1}\left(k\right){\tilde{v}}_{2|1}{\left(k\right)}^{T}\right\}\\ \; & ={\hat{v}}_{2|1}\left(k\right)E\left\{{\tilde{v}}_{2|1}{\left(k\right)}^{T}\right\}+E\left\{{\tilde{v}}_{2|1}\left(k\right){\tilde{v}}_{2|1}{\left(k\right)}^{T}\right\}\\ \; & =-{\hat{v}}_{2|1}\left(k\right){\hat{v}}_{2|1}{\left(k\right)}^{T}+E\left\{{\tilde{v}}_{2|1}\left(k\right){\tilde{v}}_{2|1}{\left(k\right)}^{T}\right\}\\ \; & \neq E\{{\tilde{v}}_{2|1}\left(k\right){\tilde{v}}_{2|1}{\left(k\right)}^{T}\} \end{array}$$

**Corollary 8.**  *The derivation of the formula $K_{2}\left(k\right)=P_{{\tilde{x}}_{1}\varepsilon_{2}^{\left(s,f\right)}}\left(k\right)Q_{\varepsilon_{2}^{\left(s,f\right)}}\left(k\right)$ contains an inconsistency, as detailed in Equation (25) of Ref. [26].*

**Proof.**  Equation (25) in Ref. [26] presents an incorrect formula for $K_{2}\left(k\right)=E\left\{x\left(k\right)\varepsilon_{2}^{\left(s,f\right)}\left(k\right)\right\}$$Q_{\varepsilon_{2}^{\left(s,f\right)}}{\left(k\right)}^{-1}$. The correct expression should be ${\bar{K}}_{2}\left(k\right)=E\left\{{\tilde{x}}_{1}\left(k\right)\varepsilon_{2}^{\left(s,r\right)}\left(k\right)\right\}\;Q_{\varepsilon_{2}^{\left(s,r\right)}}{\left(k\right)}^{-1}$. Meanwhile,

(42) $$\begin{array}{cc} \; & E\{x\left(k\right)\varepsilon_{2}^{\left(s,f\right)}{\left(k\right)}^{T}\}\\ \; & =E\{\left[{\hat{x}}_{1}\left(k\right)+{\tilde{x}}_{1}\left(k\right)\right]\varepsilon_{2}^{\left(s,f\right)}{\left(k\right)}^{T}\}\\ \; & =E\left\{{\hat{x}}_{1}\left(k\right)\varepsilon_{2}^{\left(s,f\right)}{\left(k\right)}^{T}\right\}+E\left\{{\tilde{x}}_{1}\left(k\right)\varepsilon_{2}^{\left(s,f\right)}{\left(k\right)}^{T}\right\}\\ \; & \neq E\{{\tilde{x}}_{1}\left(k\right)\varepsilon_{2}^{\left(s,f\right)}{\left(k\right)}^{T}\} \end{array}$$

□

**Corollary 9.**  *Owing to Corollary 2 and Corollary 8, Equations (37) and (38) in Ref. [26] do not hold when N=2:*

(43) $${\hat{x}}^{\left(s\right)}\left(k|k\right)\neq {\hat{x}}^{\left(s\right)}\left(k|k-1\right)+\sum_{l=1}^{2}K_{l}\left(k\right)\;\varepsilon_{l}\left(k\right)$$

(44) $$P^{\left(s\right)}\left(k|k\right)\neq P^{\left(s\right)}\left(k|k-1\right)-\sum_{l=1}^{2}K_{l}\left(k\right)\;Q_{\varepsilon_{l}\left(k\right)}\left(k\right)\;K_{l}{\left(k\right)}^{T}$$

#### 4.2.3. Analysis of the Completeness of the Global Optimality Proof for the Sequential Fusion Filter in Ref. [26]

Ref. [26] claims to prove the estimation accuracy equivalence between the proposed sequential fusion filter and the centralized fusion filter via mathematical induction. However, the proof lacks key steps and has logical gaps, making the induction incomplete and non-rigorous. Its imperfections are detailed as follows:

In the two-sensor case, Ref. [26] uses matrix block inversion to decompose the centralized innovation $\varepsilon^{\left(c\right)}\left(k\right)$ into a linear combination of the sequential innovations $\varepsilon_{1}\left(k\right)$ and $\varepsilon_{2}\left(k\right)$, as shown in the equation below:

(45) $$K^{\left(c\right)}\left(k\right)\varepsilon^{\left(c\right)}\left(k\right)=K_{1}\left(k\right)\varepsilon_{1}\left(k\right)+K_{2}\left(k\right)\varepsilon_{2}\left(k\right)$$

As analyzed in Section 4.2, the lack of clear distinction between the realized innovation and the random innovation variable results in non-orthogonal sequential innovation sequences, i.e., $E\{\varepsilon_{2}\left(k\right)\varepsilon_{1}{\left(k\right)}^{T}\}\neq 0$. Therefore, the equation derived for the two-sensor case based on this definition cannot serve as a valid basis for the subsequent induction.

Furthermore, when extending the algorithm from i−1 to *i* sensors, Ref. [26] treats the first i−1 sensors and the *i*-th sensor as two virtual sensors and directly applies the two-sensor conclusion. Nevertheless, several critical recursive steps are missing: first, the recursive derivation process is not demonstrated; second, the orthogonality among sequential innovations is not examined. For this reason, the mathematical induction is logically flawed and incomplete.

### 4.3. Research Ideas and Motivation of This Work

Ref. [26] has a reasonable overall framework yet lacks clear definitions for the basic concepts of Kalman filter design. To address this problem, this paper proposes an improved method. Using mathematical induction, we construct a new sequential fusion Kalman filter and provide a rigorous theoretical proof. The proposed filter satisfies two equivalence conditions: (a) identical filter formulations; (b) equivalent performance measured by the covariance matrix.

(1) The innovation is divided into two forms: realized innovation $\varepsilon_{i}^{\left(s,f\right)}\left(k\right)$ and random innovation variable $\varepsilon_{i}^{\left(s,r\right)}\left(k\right)$. In filter design, the random innovation variable $\varepsilon_{i}^{\left(s,r\right)}\left(k\right)$ is used, while during each execution of the filter, the realized innovation $\varepsilon_{i}^{\left(s,f\right)}\left(k\right)$ is employed. Such a differentiated application effectively avoids the problem encountered in Ref. [26].

(2) Reconstruct the measurement noise prediction estimate ${\hat{v}}_{i|i-1}\left(k\right)=\sum_{l=1}^{i-1}M_{i|l}\left(k\right)\varepsilon_{l}\left(k\right)$, which is formulated due to the lack of clear distinction between innovation concepts, and revise it into the following expressions, respectively. (a) Predict the realized innovation generated by the *i*th sensor using the previously generated realized innovations $\varepsilon_{l}^{\left(s,f\right)}\left(k\right),\;l=1,2,\cdots ,i-1$; in other words, remove the existing innovations $\varepsilon_{l}^{\left(s,f\right)}\left(k\right),\;l=1,2,\cdots ,i-1$, contained in the realized *i*th innovation of the sensor, i.e.,

(46) $$\varepsilon_{i}^{\left(s,f\right)}\left(k\right)=y_{i}\left(k\right)-H_{i}\left(k\right){\hat{x}}_{i-1}^{\left(s\right)}\left(k|k\right)-\sum_{l=1}^{i-1}M_{i|l}\left(k\right)\varepsilon_{l}^{\left(s,f\right)}\left(k\right),\;i=2,3,\ldots ,N$$

(47) $$\varepsilon_{1}^{\left(s,f\right)}\left(k\right)=y_{1}\left(k\right)-H_{1}\left(k\right)\hat{x}\left(k|k-1\right)$$

(b) Predict the random innovation variable generated by the *i*th sensor using the previously generated stochastic innovations $\varepsilon_{l}^{\left(s,r\right)}\left(k\right),\;l=1,2,\cdots ,i-1$; in other words, remove the existing innovations $\varepsilon_{l}^{\left(s,r\right)}\left(k\right),\;l=1,2,\cdots ,i-1$ contained in the random innovation variable of the *i*th, i.e.,

(48) $$\varepsilon_{i}^{\left(s,r\right)}\left(k\right)=H_{i}\left(k\right){\tilde{x}}_{i-1}^{\left(s\right)}\left(k|k\right)+v_{i}\left(k\right)-\sum_{l=1}^{i-1}M_{i|l}\left(k\right)\varepsilon_{l}^{\left(s,r\right)}\left(k\right),\;i=2,3,\ldots ,N$$

(49) $$\varepsilon_{1}^{\left(s,r\right)}\left(k\right)=H_{1}\left(k\right)\tilde{x}\left(k|k-1\right)+v_{1}\left(k\right)$$

(c) Utilize statistical orthogonality $E\{\varepsilon_{i}^{\left(s,r\right)}\left(k\right)\varepsilon_{l}^{\left(s,r\right)}{\left(k\right)}^{T}\}=0$ to solve for the system matrix $M_{i|l}\left(k\right)$.

Regarding the superscripts (s,f), (s,r) and (c,r), *s* is the abbreviation for sequential strategy and *c* for centralized strategy. Meanwhile, *f* and *r* are used to label the realized innovation and the random innovation variable, respectively.

## 5. Design of a Sequential Fusion Kalman Filter Equivalent to the Centralized Fusion Kalman Filter

In this section, we consider a linear dynamic system with dual-correlated noises, where the system process noise is correlated with all distributed measurement noises and the individual measurement noises are also correlated with each other. Aiming at the unresolved problem in existing research, we construct a sequential Kalman fusion filter that matches the centralized fusion Kalman filter in both formulation and estimation performance.

### 5.1. Summary of Verification Steps

To address the above issues, this paper constructs a statistically orthogonal sequential innovation sequence by employing a principle analogous to the Gram–Schmidt orthogonalization method. Based on this innovation sequence, a sequential fusion Kalman filter is designed using mathematical induction.

**Theorem 1.**  *For the sensor dynamic system described by Equations (1) and (2), with Equations (4) and (5) as initial conditions, a centralized Kalman fusion filter is established by Equations (6)–(14), denoted as ${\hat{x}}_{1\to i}^{\left(c\right)}\left(k|k\right)$, $P_{1\to i}^{\left(c\right)}\left(k|k\right)$. Moreover, a sequential Kalman fusion filter, denoted as ${\hat{x}}_{1\to i}^{\left(s\right)}\left(k|k\right)$, $P_{1\to i}^{\left(s\right)}\left(k|k\right)$, can be designed such that it is fully equivalent to the centralized filter in the following senses.*

Formal equivalence: The sequential filter has the same prediction-update structure as the centralized filter, i.e., ${\hat{x}}_{1\to i}^{\left(s\right)}\left(k|k\right)={\hat{x}}_{1\to i}^{\left(c\right)}\left(k|k\right)$ and the sequential innovation sequence $\varepsilon_{i}^{\left(s,r\right)}\left(k\right)$ is a linear combination of the centralized innovation $\varepsilon_{1\to i}^{\left(c,r\right)}\left(k\right)$ via an invertible transformation.

Performance equivalence: The state estimates and estimation error covariance matrices of the two filters are exactly identical, i.e., $P_{1\to i}^{\left(s\right)}\left(k|k\right)=P_{1\to i}^{\left(c\right)}\left(k|k\right)$.

Computational equivalence: The sequential filter produces identical results but avoids the inversion of high-dimensional matrices. It processes measurements one by one, requiring only the inversion of smaller matrices whose dimensions equal the measurement size of each sensor. This reduces computational complexity and memory footprint.

To this end, we assume that the distributed multi-sensor measurements arrive at the filter fusion center sequentially according to the subscript order of the sensors in the measurement system Equation (2), i.e.,

(50) $$\{y_{1}\left(k\right),y_{2}\left(k\right),\cdots ,y_{i}\left(k\right)\},\;i=1,2,\cdots ,N$$

#### 5.1.1. Verification Step 1: Establish the Sequential Fusion Filter for the Single-Sensor Case When i=1

For the linear multi-sensor system with dual-correlated noises described by Equations (1) and (2), when i=N=1, the multi-sensor system degenerates into a single-sensor system. In this case, the established centralized and sequential Kalman filters are identical in form and equivalent in performance.

At time *k*, assume that a one-step prediction is performed based on the globally optimal state estimate $\hat{x}\left(k-1|k-1\right)$ and its state estimation error covariance matrix P(k−1|k−1) at time k−1, yielding the predicted estimate $\hat{x}\left(k|k-1\right)$ and its state estimation error covariance matrix $P^{\left(s\right)}\left(k|k-1\right)$ as follows:

(51) $$\hat{x}\left(k|k-1\right)=\Phi \left(k\right)\hat{x}\left(k-1|k-1\right)$$

(52) $$P\left(k|k-1\right)=\Phi \left(k\right)P\left(k-1|k-1\right)\Phi^{T}\left(k\right)+Q_{w}\left(k\right)$$

When i=1, the sequential filter degenerates to the standard Kalman filter. Based on the observation model in Equation (2), the predicted estimate of the measurement is calculated, where the superscript “*s*” denotes the sequential filter:

(53) $${\hat{y}}_{1}^{\left(c,f\right)}\left(k|k-1\right)\equiv {\hat{y}}_{1}^{\left(s,f\right)}\left(k|k-1\right)=H_{1}\left(k\right)\hat{x}\left(k|k-1\right)$$

The deterministic innovation and stochastic innovation are given respectively by

(54) $$\begin{array}{cc} \varepsilon_{1}^{\left(c,f\right)}\left(k\right) & \equiv \varepsilon_{1}^{\left(s,f\right)}\left(k\right)=y_{1}\left(k\right)-{\hat{y}}_{1}^{\left(s\right)}\left(k|k-1\right)\\ \varepsilon_{1}^{\left(c,r\right)}\left(k\right) & \equiv \varepsilon_{1}^{\left(s,f\right)}\left(k\right)=H_{1}\left(k\right)\tilde{x}\left(k|k-1\right)+v_{1}\left(k\right) \end{array}$$

The auto-covariance matrix of the measurement prediction estimation error is

(55) $$\begin{array}{cc} Q_{\varepsilon_{1}^{\left(c,r\right)}}\left(k\right) & \equiv Q_{\varepsilon_{1}^{\left(s,r\right)}}\left(k\right)\\ \; & =E\left\{\varepsilon_{1}^{\left(s,r\right)}\left(k\right)\;\varepsilon_{1}^{\left(s,r\right)}{\left(k\right)}^{T}\right\}\\ \; & =E\left\{\left[H_{1}\left(k\right)\tilde{x}\left(k|k-1\right)+v_{1}\left(k\right)\right]{\left[H_{1}\left(k\right)\tilde{x}\left(k|k-1\right)+v_{1}\left(k\right)\right]}^{T}\right\}\\ \; & =H_{1}\left(k\right)P^{\left(s\right)}\left(k|k-1\right)H_{1}^{T}\left(k\right)+H_{1}\left(k\right)S_{1}\left(k\right)+S_{1}^{T}\left(k\right)H_{1}^{T}\left(k\right)+R_{1}\left(k\right) \end{array}$$

The cross-covariance matrix of state prediction error and measurement prediction error is

(56) $$\begin{array}{cc} P_{\tilde{x}\varepsilon_{1}^{\left(c,r\right)}}^{\left(c\right)}\left(k\right) & \equiv P_{\tilde{x}\varepsilon_{1}^{\left(c,r\right)}}^{\left(s\right)}\left(k\right)\\ \; & =E\left\{\tilde{x}\left(k|k-1\right)\;\varepsilon_{1}^{\left(s,r\right)}{\left(k\right)}^{T}\right\}\\ \; & =E\left\{\tilde{x}\left(k|k-1\right){\left[H_{1}\left(k\right)\tilde{x}\left(k|k-1\right)+v_{1}\left(k\right)\right]}^{T}\right\}\\ \; & =P\left(k|k-1\right)H_{1}^{T}\left(k\right)+S_{1}\left(k\right) \end{array}$$

Design a Kalman filter to estimate the state variable x(k):

(57) $$\begin{array}{cc} {\hat{x}}_{1}^{\left(c\right)}\left(k|k\right) & \equiv {\hat{x}}_{1}^{\left(s\right)}\left(k|k\right)\\ \; & =\hat{x}\left(k|k-1\right)+K_{1}^{\left(c\right)}\left(k\right)\varepsilon_{1}^{\left(c,f\right)}\left(k\right)\\ \; & =\hat{x}\left(k|k-1\right)+K_{1}^{\left(s\right)}\left(k\right)\varepsilon_{1}^{\left(s,f\right)}\left(k\right) \end{array}$$

The estimation error of the state variable is defined as

(58) $$\begin{array}{cc} {\tilde{x}}_{1}^{\left(c\right)}\left(k|k\right) & \equiv {\tilde{x}}_{1}^{\left(s\right)}\left(k|k\right)=x\left(k\right)-{\hat{x}}_{1}^{\left(s\right)}\left(k|k\right)\\ \; & =\tilde{x}\left(k|k-1\right)-K_{1}^{\left(c\right)}\left(k\right)\varepsilon_{1}^{\left(c,f\right)}\left(k\right)\\ \; & =\tilde{x}\left(k|k-1\right)-K_{1}^{\left(s\right)}\left(k\right)\varepsilon_{1}^{\left(s,f\right)}\left(k\right) \end{array}$$

$K_{1}^{\left(c\right)}\left(k\right)\equiv K_{1}^{\left(s\right)}\left(k\right)$ is the filter gain matrix to be identified. Using the orthogonality principle, it is derived as follows:

(59) $$\begin{array}{cc} K_{1}^{\left(c\right)}\left(k\right) & \equiv K_{1}^{\left(s\right)}\left(k\right)\\ \; & =P_{\tilde{x}\varepsilon_{1}^{\left(c,r\right)}}^{\left(c\right)}\left(k\right)\;Q_{\varepsilon_{1}^{\left(c,r\right)}}{\left(k\right)}^{-1}\\ \; & =P_{\tilde{x}\varepsilon_{1}^{\left(s,r\right)}}^{\left(s\right)}\left(k\right)\;Q_{\varepsilon_{1}^{\left(s,r\right)}}{\left(k\right)}^{-1} \end{array}$$

The estimation error covariance matrix of the state variable x(k+1) is

(60) $$\begin{array}{cc} P_{1}^{\left(c\right)}\left(k|k\right) & \equiv P_{1}^{\left(s\right)}\left(k|k\right)\\ \; & =E\left\{{\tilde{x}}_{1}^{\left(c\right)}\left(k|k\right)\;{\tilde{x}}_{1}^{\left(c\right)}{\left(k|k\right)}^{T}\right\}=E\left\{{\tilde{x}}_{1}^{\left(s\right)}\left(k|k\right)\;{\tilde{x}}_{1}^{\left(s\right)}{\left(k|k\right)}^{T}\right\}\\ \; & =P\left(k|k-1\right)-K_{1}^{\left(c\right)}\left(k\right)\;Q_{\varepsilon_{1}^{\left(c,r\right)}}\left(k\right)\;K_{1}^{\left(c\right)}{\left(k\right)}^{T}\\ \; & =P\left(k|k-1\right)-K_{1}^{\left(s\right)}\left(k\right)\;Q_{\varepsilon_{1}^{\left(s,r\right)}}\left(k\right)\;K_{1}^{\left(s\right)}{\left(k\right)}^{T} \end{array}$$

Conclusion: It is verified that when i=1, the centralized Kalman filter and the sequential Kalman filter are completely identical filters.

#### 5.1.2. Verification Step 2: Establishing the Sequential Fusion Filter for Two Sensors When i=2

Based on Sensor 2, the deterministic and stochastic innovations are respectively defined as

(61) $$\begin{array}{cc} {\bar{\varepsilon }}_{2}^{\left(s,f\right)}\left(k\right) & =y_{2}\left(k\right)-H_{2}\left(k\right){\hat{x}}_{1}^{\left(s\right)}\left(k|k\right)\\ {\bar{\varepsilon }}_{2}^{\left(s,r\right)}\left(k\right) & =H_{2}\left(k\right){\tilde{x}}_{1}^{\left(s\right)}\left(k|k\right)+v_{2}\left(k\right) \end{array}$$

Utilizing the Gram–Schmidt-like orthogonality principle, the information contained in $\varepsilon_{2}^{\left(s,f\right)}\left(k\right)$ or $\varepsilon_{2}^{\left(s,r\right)}\left(k\right)$ that overlaps with $\varepsilon_{1}^{\left(s,f\right)}\left(k\right)$ or $\varepsilon_{1}^{\left(s,r\right)}\left(k\right)$ is removed, leading to

(62) $$\begin{array}{cc} \varepsilon_{2}^{\left(s,f\right)}\left(k\right) & :={\bar{\varepsilon }}_{2}^{\left(s,f\right)}\left(k\right)-M_{2|1}\left(k\right)\varepsilon_{1}^{\left(s,f\right)}\left(k\right)\\ \; & =y_{2}\left(k\right)-H_{2}\left(k\right){\hat{x}}_{1}^{\left(s\right)}\left(k|k\right)-M_{2|1}\left(k\right)\varepsilon_{1}^{\left(s,f\right)}\left(k\right)\\ \varepsilon_{2}^{\left(s,r\right)}\left(k\right) & :={\bar{\varepsilon }}_{2}^{\left(s,r\right)}\left(k\right)-M_{2|1}\left(k\right)\varepsilon_{1}^{\left(s,r\right)}\left(k\right)\\ \; & =H_{2}\left(k\right){\tilde{x}}_{1}^{\left(s\right)}\left(k|k\right)+v_{2}\left(k\right)-M_{2|1}\left(k\right)\varepsilon_{1}^{\left(s,r\right)}\left(k\right) \end{array}$$

where the Lagrange multiplier matrix is given by

(63) $$M_{2|1}\left(k\right)=Q_{{\bar{\varepsilon }}_{2}^{\left(s,r\right)}\varepsilon_{1}^{\left(s,r\right)}}\left(k\right)Q_{\varepsilon_{1}^{\left(s,r\right)}\varepsilon_{1}^{\left(s,r\right)}}{\left(k\right)}^{-1}$$

The solution process is derived as follows:

(64) $$\begin{array}{cc} E\{\varepsilon_{2}^{\left(s,r\right)}\left(k\right)\varepsilon_{1}^{\left(s,r\right)}{\left(k\right)}^{T}\}\; & =E\{\left[{\bar{\varepsilon }}_{2}^{\left(s,r\right)}\left(k\right)-M_{2|1}\left(k\right)\varepsilon_{1}^{\left(s,r\right)}\left(k\right)\right]\varepsilon_{1}^{\left(s,r\right)}{\left(k\right)}^{T}\}\\ \; & =E\left\{{\bar{\varepsilon }}_{2}^{\left(s,r\right)}\left(k\right)\varepsilon_{1}^{\left(s,r\right)}{\left(k\right)}^{T}\right\}-M_{2|1}\left(k\right)E\left\{\varepsilon_{1}^{\left(s,r\right)}\left(k\right)\varepsilon_{1}^{\left(s,r\right)}{\left(k\right)}^{T}\right\}\\ \; & =Q_{{\bar{\varepsilon }}_{2}^{\left(s,r\right)}\varepsilon_{1}^{\left(s,r\right)}}\left(k\right)-M_{2|1}\left(k\right)Q_{\varepsilon_{1}^{\left(s,r\right)}\varepsilon_{1}^{\left(s,r\right)}}\left(k\right)=0 \end{array}$$

(65) $$\begin{array}{cc} Q_{{\bar{\varepsilon }}_{2}^{\left(s,r\right)}\varepsilon_{1}^{\left(s,r\right)}}\left(k\right) & =E\{{\bar{\varepsilon }}_{2}^{\left(s,r\right)}\left(k\right)\varepsilon_{1}^{\left(s,r\right)}{\left(k\right)}^{T}\}\\ \; & =E\{\left[H_{2}\left(k\right){\tilde{x}}_{1}^{\left(s\right)}\left(k|k\right)+v_{2}\left(k\right)\right]\varepsilon_{1}^{\left(s,r\right)}{\left(k\right)}^{T}\}\\ \; & =E\{v_{2}\left(k\right){\left[H_{1}\left(k\right)\tilde{x}\left(k|k-1\right)+v_{1}\left(k\right)\right]}^{T}\}\\ \; & =S_{2}^{T}\left(k\right)H_{1}^{T}\left(k\right)+R_{21}\left(k\right) \end{array}$$

Based on the state model and sensors 1 and 2, the centralized and sequential Kalman filters are established, and their equivalence in estimation performance is rigorously proved.

(1) Establish the centralized fusion Kalman filter: The centralized fusion estimate and the corresponding estimation error covariance matrix are given by, respectively,

(66) $$\begin{array}{cc} {\hat{x}}_{1\to 2}^{\left(c\right)}\left(k|k\right) & =E\{x\left(k\right)|\hat{x}\left(k-1|k-1\right),y_{1}\left(k\right),y_{2}\left(k\right)\}\\ \; & =\hat{x}\left(k|k-1\right)+K_{1\to 2}^{\left(c\right)}\left(k\right)\;\varepsilon_{1\to 2}^{\left(c,f\right)}\left(k\right) \end{array}$$

(67) $$\begin{array}{cc} {\tilde{x}}_{1\to 2}^{\left(c\right)}\left(k|k\right) & =x\left(k\right)-{\hat{x}}_{1\to 2}^{\left(c\right)}\left(k|k\right)\\ \; & =\tilde{x}\left(k|k-1\right)-K_{1\to 2}^{\left(c\right)}\left(k\right)\;\varepsilon_{1\to 2}^{\left(c,r\right)}\left(k\right) \end{array}$$

(68) $$\begin{array}{cc} P_{1\to 2}^{\left(c\right)}\left(k|k\right) & =E\left\{{\tilde{x}}_{1\to 2}^{\left(c\right)}\left(k|k\right)\;{\tilde{x}}_{1\to 2}^{\left(c\right)}{\left(k|k\right)}^{T}\right\}\\ \; & =P\left(k|k-1\right)-K_{1\to 2}^{\left(c\right)}\left(k\right)\;Q_{\varepsilon_{1\to 2}^{\left(c,r\right)}}\left(k\right)\;K_{1\to 2}^{\left(c\right)}{\left(k\right)}^{T} \end{array}$$

where $K_{1\to 2}^{\left(c\right)}\left(k\right)=P_{\tilde{x}\varepsilon_{1\to 2}^{\left(c,r\right)}}\left(k|k\right)\;Q_{\varepsilon_{1\to 2}^{\left(c,r\right)}}{\left(k\right)}^{-1}$,

$$P_{\tilde{x}\varepsilon_{1\to 2}^{\left(c,r\right)}}\left(k|k\right)=E\left\{\tilde{x}\left(k|k-1\right)\varepsilon_{1\to 2}^{\left(c,r\right)}(k{)}^{T}\right\}$$

$$Q_{\varepsilon_{1\to 2}^{\left(c,r\right)}(k)}(k)=E\left\{\varepsilon_{1\to 2}^{\left(c,r\right)}(k)\varepsilon_{1\to 2}^{\left(c,r\right)}(k{)}^{T}\right\}$$

$$\varepsilon_{1\to 2}^{\left(c,f\right)}(k)=\left[\begin{array}{c} \varepsilon_{1}^{\left(c,f\right)}(k)\\ \varepsilon_{2}^{\left(c,f\right)}(k) \end{array}\right]=\left[\begin{array}{c} y_{1}(k)-H_{1}\left(k\right)\hat{x}\left(k|k-1\right)\\ y_{2}(k)-H_{2}\left(k\right)\hat{x}\left(k|k-1\right) \end{array}\right]$$

$$\varepsilon_{1\to 2}^{\left(c,r\right)}(k)=\left[\begin{array}{c} \varepsilon_{1}^{\left(c,r\right)}(k)\\ \varepsilon_{2}^{\left(c,r\right)}(k) \end{array}\right]=\left[\begin{array}{c} H_{1}\left(k\right)\tilde{x}(k|k-1)+v_{1}(k)\\ H_{2}\left(k\right)\tilde{x}(k|k-1)+v_{2}(k) \end{array}\right]$$

(2) Establish the sequential fusion Kalman filter: The sequential fused state estimate and the corresponding estimation error covariance matrix are given by, respectively,

(69) $$\begin{array}{cc} {\hat{x}}_{2}^{\left(s\right)}\left(k\right) & ={\hat{x}}_{1}^{\left(s\right)}\left(k|k\right)+K_{2}^{\left(s\right)}\left(k\right)\varepsilon_{2}^{\left(s,f\right)}\left(k\right)\\ \; & ={\hat{x}}^{\left(s\right)}\left(k|k-1\right)+K_{1}^{\left(s\right)}\left(k\right)\varepsilon_{1}^{\left(s,f\right)}\left(k\right)+K_{2}^{\left(s\right)}\left(k\right)\varepsilon_{2}^{\left(s,f\right)}\left(k\right) \end{array}$$

(70) $$\begin{array}{cc} {\tilde{x}}_{2}^{\left(s\right)}\left(k|k\right) & =x\left(k\right)-{\hat{x}}_{2}^{\left(s\right)}\left(k|k\right)\\ \; & ={\tilde{x}}_{1}^{\left(s\right)}\left(k|k\right)-K_{2}^{\left(s\right)}\left(k\right)\varepsilon_{2}^{\left(s,r\right)}\left(k\right)\\ \; & =\tilde{x}\left(k|k-1\right)-K_{1}^{\left(s\right)}\left(k\right)\varepsilon_{1}^{\left(s,r\right)}\left(k\right)-K_{2}^{\left(s\right)}\left(k\right)\varepsilon_{2}^{\left(s,r\right)}\left(k\right) \end{array}$$

(71) $$\begin{array}{cc} P_{2}^{\left(s\right)}\left(k|k\right) & =E\{{\tilde{x}}_{2}^{\left(s\right)}\left(k|k\right){\tilde{x}}_{2}^{\left(s\right)}{\left(k|k\right)}^{T}\}\\ \; & =P_{1}^{\left(s\right)}\left(k|k\right)-K_{2}^{\left(s\right)}\left(k\right)Q_{\varepsilon_{2}^{\left(s,r\right)}}\left(k\right)K_{2}^{\left(s\right)}{\left(k\right)}^{T}\\ \; & =P\left(k|k-1\right)-K_{1}^{\left(s\right)}\left(k\right)Q_{\varepsilon_{1}^{\left(s,r\right)}}\left(k\right)K_{1}^{\left(s\right)}{\left(k\right)}^{T}-K_{2}^{\left(s\right)}\left(k\right)Q_{\varepsilon_{2}^{\left(s,r\right)}}\left(k\right)K_{2}^{\left(s\right)}{\left(k\right)}^{T} \end{array}$$

(72) $$K_{2}^{\left(s\right)}\left(k\right)=P_{{\tilde{x}}_{1}\varepsilon_{2}^{\left(s,r\right)}}\left(k|k-1\right)\;Q_{\varepsilon_{2}^{\left(s,r\right)}}{\left(k\right)}^{-1}$$

where

(73) $$\begin{array}{cc} P_{{\tilde{x}}_{1}\varepsilon_{1}^{\left(s,r\right)}}\left(k|k-1\right) & =E\{{\tilde{x}}_{1}\left(k|k\right)\;\varepsilon_{2}^{\left(s,r\right)}{\left(k\right)}^{T}\}\\ \; & =E\{{\tilde{x}}_{1}\left(k|k\right)\;{\bar{\varepsilon }}_{2}^{\left(s,r\right)}{\left(k\right)}^{T}\}\\ \; & =P_{1}^{\left(s\right)}\left(k|k\right)H_{2}^{T}\left(k\right)+P_{{\tilde{x}}_{1}v_{2}}\left(k\right) \end{array}$$

(74) $$\begin{array}{cc} Q_{\varepsilon_{2}^{\left(s,r\right)}}\left(k\right) & =E\{\varepsilon_{2}^{\left(s,r\right)}\left(k\right)\;\varepsilon_{2}^{\left(s,r\right)}{\left(k\right)}^{T}\}\\ \; & =E\{\left[{\bar{\varepsilon }}_{2}^{\left(s,r\right)}\left(k\right)-M_{2|1}\left(k\right)\varepsilon_{1}^{\left(s,r\right)}\left(k\right)\right]{\left[{\bar{\varepsilon }}_{2}^{\left(s,r\right)}\left(k\right)-M_{2|1}\left(k\right)\varepsilon_{1}^{\left(s,r\right)}\left(k\right)\right]}^{T}\}\\ \; & =E\left\{{\bar{\varepsilon }}_{2}^{\left(s,r\right)}\left(k\right){\bar{\varepsilon }}_{2}^{\left(s,r\right)}{\left(k\right)}^{T}\right\}-Q_{{\bar{\varepsilon }}_{2}^{\left(s,r\right)}\varepsilon_{1}^{\left(s,r\right)}}\left(k\right)M_{2|1}^{T}\left(k\right)\\ \; & =H_{2}\left(k\right)P_{1}\left(k|k\right)H_{2}^{T}\left(k\right)+H_{2}\left(k\right)P_{{\tilde{x}}_{1}v_{2}}\left(k\right)+P_{{\tilde{x}}_{1}v_{2}}^{T}\left(k\right)H_{2}^{T}\left(k\right)\\ \; & \;+R_{2}\left(k\right)-Q_{{\bar{\varepsilon }}_{2}^{\left(s,r\right)}\varepsilon_{1}^{\left(s,r\right)}}\left(k\right)M_{2|1}^{T}\left(k\right) \end{array}$$

(75) $$\begin{array}{cc} P_{{\tilde{x}}_{1}v_{2}}\left(k\right) & =E\{{\tilde{x}}_{1}\left(k|k\right)\;v_{2}{\left(k\right)}^{T}\}\\ \; & =E\{\left[\tilde{x}\left(k|k-1\right)-K_{1}^{\left(s\right)}\left(k\right)\varepsilon_{1}^{\left(s,r\right)}\left(k\right)\right]\;v_{2}{\left(k\right)}^{T}\}\\ \; & =S_{2}\left(k\right)-K_{1}^{\left(s\right)}\left(k\right)Q_{{\bar{\varepsilon }}_{2}^{\left(s,r\right)}\varepsilon_{1}^{\left(s,r\right)}}^{T}\left(k\right) \end{array}$$

(3) Equivalence analysis between the innovations of the centralized filter and the sequential filter:

Owing to

(76) $$\begin{array}{cc} \varepsilon_{2}^{\left(s,r\right)}\left(k\right) & ={\bar{\varepsilon }}_{2}^{\left(s,r\right)}\left(k\right)-M_{2|1}\left(k\right)\varepsilon_{1}^{\left(s,r\right)}\left(k\right)\\ \; & =H_{2}\left(k\right){\tilde{x}}_{1}^{\left(s\right)}\left(k|k\right)+v_{2}\left(k\right)-M_{2|1}\left(k\right)\varepsilon_{1}^{\left(s,r\right)}\left(k\right)\\ \; & =H_{2}\left(k\right)\left[\tilde{x}\left(k|k-1\right)-K_{1}^{\left(s\right)}\left(k\right)\varepsilon_{1}^{\left(s,r\right)}\left(k\right)\right]+v_{2}\left(k\right)-M_{2|1}\left(k\right)\varepsilon_{1}^{\left(s,r\right)}\left(k\right)\\ \; & =H_{2}\left(k\right)\tilde{x}\left(k|k-1\right)+v_{2}\left(k\right)-H_{2}\left(k\right)K_{1}^{\left(s\right)}\left(k\right)\varepsilon_{1}^{\left(s,r\right)}\left(k\right)-M_{2|1}\left(k\right)\varepsilon_{1}^{\left(s,r\right)}\left(k\right)\\ \; & =\varepsilon_{2}^{\left(c,r\right)}\left(k\right)-\left[H_{2}\left(k\right)K_{1}^{\left(s\right)}\left(k\right)+M_{2|1}\left(k\right)\right]\varepsilon_{1}^{\left(s,r\right)}\left(k\right) \end{array}$$

(77) $$\varepsilon_{2}^{\left(c,r\right)}\left(k\right)=\varepsilon_{2}^{\left(s,r\right)}\left(k\right)+{\bar{M}}_{2|1}\left(k\right)\varepsilon_{1}^{\left(s,r\right)}\left(k\right)$$

We derive the equivalence relation between the innovations of the centralized and sequential filters.

(78) $$\varepsilon_{1\to 2}^{\left(c,r\right)}\left(k\right)=\left[\begin{array}{c} \varepsilon_{1}^{\left(c,r\right)}\left(k\right)\\ \varepsilon_{2}^{\left(c,r\right)}\left(k\right) \end{array}\right]=M_{2,2}^{\left(c,s\right)}\left(k\right)\left[\begin{array}{c} \varepsilon_{1}^{\left(s,r\right)}\left(k\right)\\ \varepsilon_{2}^{\left(s,r\right)}\left(k\right) \end{array}\right]$$

(79) $$\varepsilon_{1\to 2}^{\left(c,f\right)}\left(k\right)=\left[\begin{array}{c} \varepsilon_{1}^{\left(c,f\right)}\left(k\right)\\ \varepsilon_{2}^{\left(c,f\right)}\left(k\right) \end{array}\right]=M_{2,2}^{\left(c,s\right)}\left(k\right)\left[\begin{array}{c} \varepsilon_{1}^{\left(s,f\right)}\left(k\right)\\ \varepsilon_{2}^{\left(s,f\right)}\left(k\right) \end{array}\right]$$

where $|M_{2,2}^{\left(c,s\right)}\left(k\right)|\neq 0$, $M_{2,2}^{\left(c,s\right)}\left(k\right)=\left[\begin{array}{cc} I_{1} & 0\\ {\bar{M}}_{2|1}\left(k\right) & I_{2} \end{array}\right]$, ${\bar{M}}_{2|1}\left(k\right)=H_{2}\left(k\right)K_{1}^{\left(s\right)}\left(k\right)+M_{2|1}\left(k\right)$.

(4) Equivalence analysis of centralized and sequential innovation gains:

The equivalence relation between the gain matrix of the centralized filter and the gain matrix of the sequential filter:

(80) $$\begin{array}{cc} K_{1\to 2}^{\left(c\right)}\left(k\right) & =P_{\tilde{x}\varepsilon_{1\to 2}^{\left(c,r\right)}}\left(k|k-1\right)\;Q_{\varepsilon_{1\to 2}^{\left(c,r\right)}}{\left(k\right)}^{-1}\\ \; & =E\left\{\tilde{x}\left(k|k-1\right)\;\varepsilon_{1\to 2}^{\left(c,r\right)}{\left(k\right)}^{T}\right\}\;E{\left\{\varepsilon_{1\to 2}^{\left(c,r\right)}\left(k\right)\;\varepsilon_{1\to 2}^{\left(c,r\right)}{\left(k\right)}^{T}\right\}}^{-1}\\ \; & =E\left\{\tilde{x}\left(k|k-1\right){\left[\begin{array}{c} \varepsilon_{1}^{\left(s,r\right)}\left(k\right)\\ \varepsilon_{2}^{\left(s,r\right)}\left(k\right) \end{array}\right]}^{T}\right\}M_{2,2}^{\left(c,s\right)}{\left(k\right)}^{T}\\ \; & \;\times M_{2,2}^{\left(c,s\right)}{\left(k\right)}^{-T}\;E{\left\{\left[\begin{array}{c} \varepsilon_{1}^{\left(s,r\right)}\left(k\right)\\ \varepsilon_{2}^{\left(s,r\right)}\left(k\right) \end{array}\right]{\left[\begin{array}{c} \varepsilon_{1}^{\left(s,r\right)}\left(k\right)\\ \varepsilon_{2}^{\left(s,r\right)}\left(k\right) \end{array}\right]}^{T}\right\}}^{-1}M_{2,2}^{\left(c,s\right)}{\left(k\right)}^{-1}\\ \; & =E\left\{\tilde{x}\left(k|k-1\right)\varepsilon_{1}^{\left(s,r\right)}{\left(k\right)}^{T},\;\tilde{x}\left(k|k-1\right)\varepsilon_{2}^{\left(s,r\right)}{\left(k\right)}^{T}\right\}\left[\begin{array}{cc} Q_{\varepsilon_{1}^{\left(s,r\right)}}{\left(k\right)}^{-1} & 0\\ 0 & Q_{\varepsilon_{2}^{\left(s,r\right)}}{\left(k\right)}^{-1} \end{array}\right]M_{2,2}^{\left(c,s\right)}{\left(k\right)}^{-1}\\ \; & =\left[\begin{array}{cc} P_{\tilde{x}\varepsilon_{1}^{\left(s,r\right)}}\left(k\right) & P_{\tilde{x}\varepsilon_{2}^{\left(s,r\right)}}\left(k\right) \end{array}\right]\left[\begin{array}{cc} Q_{\varepsilon_{1}^{\left(s,r\right)}}{\left(k\right)}^{-1} & 0\\ 0 & Q_{\varepsilon_{2}^{\left(s,r\right)}}{\left(k\right)}^{-1} \end{array}\right]M_{2,2}^{\left(c,s\right)}{\left(k\right)}^{-1}\\ \; & =\left[\begin{array}{cc} K_{1}^{\left(s\right)}\left(k\right) & K_{2}^{\left(s\right)}\left(k\right) \end{array}\right]M_{2,2}^{\left(c,s\right)}{\left(k\right)}^{-1} \end{array}$$

(81) $$\begin{array}{cc} K_{1\to 2}^{\left(c\right)}\left(k\right)\;\varepsilon_{1\to 2}^{\left(c,f\right)}\left(k\right)\; & =\left[K_{1}^{\left(s\right)}\left(k\right),\;K_{2}^{\left(s\right)}\left(k\right)\right]\;M_{2,2}^{\left(c,s\right)}{\left(k\right)}^{-1}\;M_{2,2}^{\left(c,s\right)}\left(k\right)\left[\begin{array}{c} \varepsilon_{1}^{\left(s,f\right)}\left(k\right)\\ \varepsilon_{2}^{\left(s,f\right)}\left(k\right) \end{array}\right]\\ \; & =K_{1}^{\left(s\right)}\left(k\right)\;\varepsilon_{1}^{\left(s,f\right)}\left(k\right)+K_{2}^{\left(s\right)}\left(k\right)\;\varepsilon_{2}^{\left(s,f\right)}\left(k\right) \end{array}$$

Among which, by employing the orthogonality principle, $E\{\varepsilon_{1}^{\left(s,r\right)}\left(k\right)\varepsilon_{2}^{\left(s,r\right)}{\left(k\right)}^{T}\}=0$.

(5) The estimation error covariance matrix of the centralized filter is identical to that of the sequential filter:

(82) $$\begin{array}{c} K_{1\to 2}^{\left(c\right)}(k)Q_{\varepsilon_{1\to 2}^{\left(c,r\right)}}(k)K_{1\to 2}^{\left(c\right)}(k{)}^{T}=\left[K_{1}^{\left(s\right)}\left(k\right),K_{2}^{\left(s\right)}\left(k\right)\right]M_{2,2}^{\left(c,s\right)}{\left(k\right)}^{-1}\\ \times M_{2,2}^{\left(c,s\right)}\left(k\right)E\left\{\left[\begin{array}{c} \varepsilon_{1}^{\left(s,r\right)}\left(k\right)\\ \varepsilon_{2}^{\left(s,r\right)}\left(k\right) \end{array}\right]{\left[\begin{array}{c} \varepsilon_{1}^{\left(s,r\right)}\left(k\right)\\ \varepsilon_{2}^{\left(s,r\right)}\left(k\right) \end{array}\right]}^{T}\right\}M_{2,2}^{\left(c,s\right)}{\left(k\right)}^{T}{\left[\left[K_{1}^{\left(s\right)}\left(k\right),K_{2}^{\left(s\right)}\left(k\right)\right]M_{2,2}^{\left(c,s\right)}{\left(k\right)}^{-1}\right]}^{T}\\ =\left[K_{1}^{\left(s\right)}\left(k\right),K_{2}^{\left(s\right)}\left(k\right)\right]\left[\begin{array}{cc} Q_{\varepsilon_{1}^{\left(s,r\right)}}(k) & 0\\ 0 & Q_{\varepsilon_{2}^{\left(s,r\right)}}(k) \end{array}\right]M_{2,2}^{\left(c,s\right)}{\left(k\right)}^{T}M_{2,2}^{\left(c,s\right)}{\left(k\right)}^{-T}\left[\begin{array}{c} K_{1}^{\left(s\right)}{\left(k\right)}^{T}\\ K_{2}^{\left(s\right)}{\left(k\right)}^{T} \end{array}\right]\\ =K_{1}^{\left(s\right)}\left(k\right)Q_{\varepsilon_{1}^{\left(s,r\right)}}(k)K_{1}^{\left(s\right)}{\left(k\right)}^{T}+K_{2}^{\left(s\right)}\left(k\right)Q_{\varepsilon_{2}^{\left(s,r\right)}}(k)K_{2}^{\left(s\right)}{\left(k\right)}^{T} \end{array}$$

### 5.2. Inductive Hypothesis Step

For i>2, a sequential Kalman fusion filter is developed based on the sequentially arriving and mutually orthogonal innovation vectors.

(1) Construct the Kalman filter for centralized fusion:

(83) $$\begin{array}{cc} {\hat{x}}_{1\to i}^{\left(c\right)}\left(k|k\right) & =E\{x\left(k\right)|\hat{x}\left(k-1|k-1\right),y_{1}\left(k\right),y_{2}\left(k\right),\ldots ,y_{i}\left(k\right)\}\\ \; & =\hat{x}\left(k|k-1\right)+K_{1\to i}^{\left(c\right)}\left(k\right)\;\varepsilon_{1\to i}^{\left(c,f\right)}\left(k\right) \end{array}$$

$${\tilde{x}}_{1\to i}^{\left(c\right)}(k|k)=\tilde{x}(k|k-1)-K_{1\to i}^{\left(c\right)}(k)\varepsilon_{1\to i}^{\left(c,r\right)}(k)$$

(84) $$\begin{array}{cc} P_{1\to i}^{\left(c\right)}\left(k|k\right) & =E\left\{{\tilde{x}}_{1\to i}^{\left(c\right)}\left(k|k\right)\;{\tilde{x}}_{1\to i}^{\left(c\right)}{\left(k|k\right)}^{T}\right\}\\ \; & =P\left(k|k-1\right)-K_{1\to i}^{\left(c\right)}\left(k\right)\;Q_{\varepsilon_{1\to i}^{\left(c,r\right)}}\left(k\right)\;K_{1\to i}^{\left(c\right)}{\left(k\right)}^{T} \end{array}$$

where $K_{1\to i}^{\left(c\right)}\left(k\right)=E\left\{\tilde{x}\left(k|k-1\right)\;\varepsilon_{1\to i}^{\left(c,r\right)}{\left(k\right)}^{T}\right\}\;E{\left\{\varepsilon_{1\to i}^{\left(c,r\right)}\left(k\right)\;\varepsilon_{1\to i}^{\left(c,r\right)}{\left(k\right)}^{T}\right\}}^{-1}=P_{\tilde{x}\varepsilon_{1\to i}^{\left(c,r\right)}}\left(k\right)\;Q_{\varepsilon_{1\to i}^{\left(c,r\right)}}{\left(k\right)}^{-1}$.

$$\varepsilon_{1\to i}^{\left(c,f\right)}(k)=\left[\begin{array}{c} \varepsilon_{1}^{\left(c,f\right)}(k)\\ \varepsilon_{2}^{\left(c,f\right)}(k)\\ \vdots \\ \varepsilon_{i}^{\left(c,f\right)}(k) \end{array}\right]=\left[\begin{array}{c} y_{1}(k)-H_{1}\left(k\right)\hat{x}\left(k|k-1\right)\\ y_{2}(k)-H_{2}\left(k\right)\hat{x}\left(k|k-1\right)\\ \vdots \\ y_{i}(k)-H_{i}\left(k\right)\hat{x}\left(k|k-1\right) \end{array}\right]$$

$$\varepsilon_{1\to i}^{\left(c,r\right)}(k)=\left[\begin{array}{c} \varepsilon_{1}^{\left(c,r\right)}(k)\\ \varepsilon_{2}^{\left(c,r\right)}(k)\\ \vdots \\ \varepsilon_{i}^{\left(c,r\right)}(k) \end{array}\right]=\left[\begin{array}{c} H_{1}\left(k\right)\tilde{x}(k|k-1)+v_{1}(k)\\ H_{2}\left(k\right)\tilde{x}(k|k-1)+v_{2}(k)\\ \vdots \\ H_{i}\left(k\right)\tilde{x}(k|k-1)+v_{i}(k) \end{array}\right]$$

(2) Construct the sequential fusion Kalman filter:

(85) $${\hat{x}}_{i}^{\left(s\right)}\left(k\right)={\hat{x}}^{\left(s\right)}\left(k|k-1\right)+\sum_{j=1}^{i}K_{j}^{\left(s\right)}\left(k\right)\;\varepsilon_{j}^{\left(s,f\right)}\left(k\right)={\hat{x}}^{\left(s\right)}\left(k|k-1\right)+K_{1\to i}^{\left(s\right)}\left(k\right)\;\varepsilon_{1\to i}^{\left(s,f\right)}\left(k\right)$$

(86) $$\begin{array}{cc} P_{1\to i}^{\left(s\right)}\left(k|k\right) & =E\left\{{\tilde{x}}_{i}^{\left(s\right)}\left(k|k\right)\;{\tilde{x}}_{i}^{\left(s\right)}{\left(k|k\right)}^{T}\right\}\\ \; & =P\left(k|k-1\right)-K_{1\to i}^{\left(s\right)}\left(k\right)\;Q_{\varepsilon_{1\to i}^{\left(s,r\right)}}\left(k\right)\;K_{1\to i}^{\left(s\right)}{\left(k\right)}^{T}\\ \; & =P\left(k|k-1\right)-\sum_{j=1}^{i}K_{j}^{\left(s\right)}\left(k\right)\;Q_{\varepsilon_{j}^{\left(s,r\right)}}\left(k\right)\;K_{j}^{\left(s\right)}{\left(k\right)}^{T} \end{array}$$

where

$$K_{1\to i}^{\left(s\right)}\left(k\right)=\left[K_{1}^{\left(s\right)}\left(k\right),\cdots ,K_{j}^{\left(s\right)}\left(k\right),\cdots ,K_{i}^{\left(s\right)}\left(k\right)\right]$$

$$\varepsilon_{1\to i}^{\left(s,f\right)}(k)={\left[\varepsilon_{1}^{\left(s,f\right)}(k{)}^{T},\cdots ,\varepsilon_{j}^{\left(s,f\right)}(k{)}^{T},\cdots ,\varepsilon_{i}^{\left(s,f\right)}(k{)}^{T}\right]}^{T}$$

$$\varepsilon_{j}^{\left(s,f\right)}(k)=H_{j}\left(k\right){\hat{x}}_{j-1}\left(k|k\right)-\sum_{l=1}^{j}K_{l}^{\left(s\right)}\left(k\right)\varepsilon_{l}^{\left(s,f\right)}(k)$$

$$K_{j}^{\left(s\right)}\left(k\right)=P_{{\tilde{x}}_{j-1}\varepsilon_{j}^{\left(s,r\right)}}\left(k|k-1\right)Q_{\varepsilon_{j}^{\left(s,r\right)}}{\left(k\right)}^{-1};j=1,2,\cdots ,i;{\tilde{x}}_{0}\left(k\right)=\tilde{x}\left(k|k-1\right)$$

$$\begin{array}{c} {\tilde{x}}_{i}^{\left(s\right)}\left(k|k\right)=x\left(k\right)-{\hat{x}}_{i}^{\left(s\right)}\left(k|k\right)\\ =\tilde{x}(k|k-1)-\sum_{j=1}^{i}K_{j}^{\left(s\right)}\left(k\right)\varepsilon_{j}^{\left(s,r\right)}(k)\\ =\tilde{x}(k|k-1)-K_{1\to i}^{\left(s\right)}\left(k\right)\varepsilon_{1\to i}^{\left(s,r\right)}(k) \end{array}$$

$$\begin{array}{c} \varepsilon_{j}^{\left(s,r\right)}(k)={\bar{\varepsilon }}_{j}^{\left(s,r\right)}(k)-\sum_{l=1}^{j}K_{l}^{\left(s\right)}\left(k\right)\varepsilon_{l}^{\left(s,r\right)}(k)\\ \;\;\;\;\;\;\;\;\;\;\;\;\;=H_{j}\left(k\right){\tilde{x}}_{j-1}\left(k|k\right)+v_{j}\left(k\right)-\sum_{l=1}^{j}K_{l}^{\left(s\right)}\left(k\right)\varepsilon_{l}^{\left(s,r\right)}(k) \end{array}$$

(3) Equivalence analysis between the centralized filter innovation and the sequential innovation:

(87) $$\varepsilon_{1\to i}^{\left(c,f\right)}\left(k\right)=M_{i,i}^{\left(c,s\right)}\left(k\right)\;\varepsilon_{1\to i}^{\left(s,f\right)}\left(k\right),\;\varepsilon_{1\to i}^{\left(c,r\right)}\left(k\right)=M_{i,i}^{\left(c,s\right)}\left(k\right)\;\varepsilon_{1\to i}^{\left(s,r\right)}\left(k\right)$$

(88) $$M_{i,i}^{\left(c,s\right)}\left(k\right)=\left[\begin{array}{cccc} I_{1} & 0 & \cdots & 0\\ {\bar{M}}_{2|1}\left(k\right) & I_{2} & \cdots & 0\\ \vdots & \vdots & \ddots & \vdots \\ {\bar{M}}_{i|1}\left(k\right) & {\bar{M}}_{i|2}\left(k\right) & \cdots & I_{i} \end{array}\right],\;\left|M_{i,i}^{\left(c,s\right)}\left(k\right)\right|\neq 0$$

(4) Equivalence between the innovation gain of the centralized filter and the sequential innovation gain:

(89) $$K_{1\to i}^{\left(c\right)}\left(k\right)\;\varepsilon_{1\to i}^{\left(c,f\right)}\left(k\right)=\sum_{j=1}^{i}K_{j}^{\left(s\right)}\left(k\right)\;\varepsilon_{j}^{\left(s,r\right)}\left(k\right)=K_{1\to i}^{\left(s\right)}\left(k\right)\;\varepsilon_{1\to i}^{\left(s,f\right)}\left(k\right)$$

(5) The estimation error covariance matrix of the centralized filter is identical to that of the sequential filter:

(90) $$K_{1\to i}^{\left(c\right)}\left(k\right)\;Q_{\varepsilon_{1\to i}^{\left(c,r\right)}}\left(k\right)\;K_{1\to i}^{\left(c\right)}{\left(k\right)}^{T}=\sum_{j=1}^{i}K_{j}^{\left(s\right)}\left(k\right)\;Q_{\varepsilon_{j}^{\left(s,r\right)}}\left(k\right)\;K_{j}^{\left(s\right)}{\left(k\right)}^{T}=K_{1\to i}^{\left(s\right)}\left(k\right)\;Q_{\varepsilon_{1\to i}^{\left(s,r\right)}}\left(k\right)\;K_{1\to i}^{\left(s\right)}{\left(k\right)}^{T}$$

### 5.3. Inductive Reasoning for Recursive Sequential Kalman Filter: Extending from *i* Sensors to i+1 Sensors

(1) Construct the Kalman filter for centralized fusion:

(91) $$\begin{array}{cc} {\hat{x}}_{i+1}^{\left(c\right)}\left(k|k\right) & =E\{x\left(k\right)|\hat{x}\left(k-1|k-1\right),y_{1}\left(k\right),y_{2}\left(k\right),\ldots ,y_{i}\left(k\right),y_{i+1}\left(k\right)\}\\ \; & =\hat{x}\left(k|k-1\right)+K_{1\to i+1}^{\left(c\right)}\left(k|k\right)\;\varepsilon_{1\to i+1}^{\left(c,f\right)}\left(k\right) \end{array}$$

$${\tilde{x}}_{i+1}^{\left(c\right)}(k|k)=\tilde{x}(k|k-1)-K_{1\to i+1}^{\left(c\right)}(k)\varepsilon_{1\to i+1}^{\left(c,r\right)}(k)$$

(92) $$\begin{array}{cc} P_{i+1}^{\left(c\right)}\left(k|k\right) & =E\left\{{\tilde{x}}_{i+1}^{\left(c\right)}\left(k|k\right)\;{\tilde{x}}_{i+1}^{\left(c\right)}{\left(k|k\right)}^{T}\right\}\\ \; & =P\left(k|k-1\right)-K_{1\to i+1}^{\left(c\right)}\left(k\right)\;Q_{\varepsilon_{1\to i+1}^{\left(c,r\right)}}\left(k\right)\;K_{1\to i+1}^{\left(c\right)}{\left(k\right)}^{T} \end{array}$$

where

$$K_{1\to i+1}^{\left(c\right)}\left(k\right)=E\left\{\tilde{x}\left(k|k-1\right)\;\varepsilon_{1\to i+1}^{(c,r)T}\right\}\;E{\left\{\varepsilon_{1\to i+1}^{\left(c,r\right)}\left(k\right)\;\varepsilon_{1\to i+1}^{\left(c,r\right)}{\left(k\right)}^{T}\right\}}^{-1}=P_{\tilde{x}\varepsilon_{1\to i+1}^{\left(c,r\right)}}\left(k\right)\;Q_{\varepsilon_{1\to i+1}^{\left(c,r\right)}}{\left(k\right)}^{-1}$$

$$\varepsilon_{1\to i+1}^{\left(c,f\right)}(k)=\left[\begin{array}{c} \varepsilon_{1\to i}^{\left(c,f\right)}(k)\\ \varepsilon_{i+1}^{\left(c,f\right)}(k) \end{array}\right],\varepsilon_{i+1}^{\left(c,f\right)}(k)=y_{i+1}(k)-H_{i+1}\left(k\right)\hat{x}\left(k|k-1\right)$$

$$\varepsilon_{1\to i+1}^{\left(c,r\right)}(k)=\left[\begin{array}{c} \varepsilon_{1\to i}^{\left(c,r\right)}(k)\\ \varepsilon_{i+1}^{\left(c,r\right)}(k) \end{array}\right],\varepsilon_{i+1}^{\left(c,r\right)}(k)=H_{i+1}\left(k\right)\tilde{x}(k|k-1)+v_{i+1}\left(k\right)$$

(2) Construct the sequential fusion Kalman filter:

(93) $$\varepsilon_{i+1}^{\left(s,f\right)}\left(k\right)=y_{i+1}\left(k\right)-H_{i+1}\left(k\right)\;{\hat{x}}_{i}^{\left(s\right)}\left(k|k\right)-\sum_{j=1}^{i}M_{i+1|j}\left(k\right)\;\varepsilon_{j}^{\left(s,f\right)}\left(k\right)$$

(94) $$\begin{array}{cc} \varepsilon_{i+1}^{\left(s,r\right)}\left(k\right) & ={\bar{\varepsilon }}_{i+1}^{\left(s,r\right)}\left(k\right)-\sum_{j=1}^{i}M_{i+1|j}\left(k\right)\;\varepsilon_{j}^{\left(s,r\right)}\left(k\right)\\ \; & =H_{i+1}\left(k\right)\;{\tilde{x}}_{i}^{\left(s\right)}\left(k|k\right)+v_{i+1}\left(k\right)-\sum_{j=1}^{i}M_{i+1|j}\left(k\right)\;\varepsilon_{j}^{\left(s,r\right)}\left(k\right) \end{array}$$

Using statistical orthogonality, $E\{\varepsilon_{i+1}^{\left(s,r\right)}\left(k\right)\;\varepsilon_{j}^{\left(s,r\right)}{\left(k\right)}^{T}\}=0$,

(95) $$M_{i+1|j}\left(k\right)=Q_{{\bar{\varepsilon }}_{i+1}^{\left(s,r\right)}\varepsilon_{j}^{\left(s,r\right)}}\left(k\right)\;Q_{\varepsilon_{j}^{\left(s,r\right)}\varepsilon_{j}^{\left(s,r\right)}}{\left(k\right)}^{-1},\;j=1,2,\ldots ,i$$

where

$$\begin{array}{c} Q_{{\bar{\varepsilon }}_{i+1}^{\left(s,r\right)}\varepsilon_{j}^{\left(s,r\right)}}\left(k\right)=E\left\{{\bar{\varepsilon }}_{i+1}^{\left(s,r\right)}\left(k\right)\varepsilon_{j}^{\left(s,r\right)}{\left(k\right)}^{T}\right\}\\ \;\;\;\;\;\;\;\;\;\;\;\;\;\;\;\;\;\;\;\;=E\{v_{i+1}\left(k\right)\varepsilon_{j}^{\left(s,r\right)}{\left(k\right)}^{T}\}\\ \;\;\;\;\;\;\;\;\;\;\;\;\;\;\;\;\;\;\;\;=E\{v_{i+1}\left(k\right){\left[H_{j}\left(k\right){\tilde{x}}_{j-1}^{\left(s\right)}(k|k)+v_{j}\left(k\right)-\sum_{q=1}^{j-1}M_{j|q}\left(k\right)\varepsilon_{q}^{\left(s,r\right)}\left(k\right)\right]}^{T}\}\\ \;\;\;\;\;\;\;\;\;\;\;\;\;\;\;\;\;\;\;\;=P_{{\tilde{x}}_{i}v_{i+1}}{\left(k\right)}^{T}H_{j}^{T}\left(k\right)+R_{i+1j}\left(k\right)-\sum_{q=1}^{j-1}Q_{{\bar{\varepsilon }}_{i+1}^{\left(s,r\right)}\varepsilon_{q}^{\left(s,r\right)}}\left(k\right)M_{j|q}^{T}\left(k\right) \end{array}$$

$$\begin{array}{c} P_{{\tilde{x}}_{i}v_{i+1}}\left(k\right)=E\left\{{\tilde{x}}_{i}^{\left(s\right)}(k|k\right)v_{i+1}{\left(k\right)}^{T}\}\\ =E\{\left[\tilde{x}(k|k-1)-\sum_{j=1}^{i}K_{j}^{\left(s\right)}\left(k\right)\varepsilon_{j}^{\left(s,r\right)}(k)\right]v_{i+1}{\left(k\right)}^{T}\}\\ =S_{i+1}\left(k\right)-\sum_{j=1}^{i}K_{j}^{\left(s\right)}\left(k\right)Q_{{\bar{\varepsilon }}_{i+1}^{\left(s,r\right)}\varepsilon_{j}^{\left(s,r\right)}}{\left(k\right)}^{T} \end{array}$$

The sequential fused state estimate and the corresponding estimation error covariance matrix are given by, respectively:

(96) $$\begin{array}{cc} {\hat{x}}_{1\to i+1}^{\left(s\right)}\left(k|k\right) & =\hat{x}\left(k|k-1\right)+\sum_{j=1}^{i}K_{j}^{\left(s\right)}\left(k\right)\;\varepsilon_{j}^{\left(s,f\right)}\left(k\right)+K_{i+1}^{\left(s\right)}\left(k\right)\;\varepsilon_{i+1}^{\left(s,f\right)}\left(k\right)\\ \; & =\hat{x}\left(k|k-1\right)+K_{1\to i}^{\left(s\right)}\left(k\right)\;\varepsilon_{1\to i}^{\left(s,f\right)}{\left(k\right)}^{T}+K_{i+1}^{\left(s\right)}\left(k\right)\;\varepsilon_{i+1}^{\left(s,f\right)}\left(k\right) \end{array}$$

(97) $$\begin{array}{cc} {\tilde{x}}_{1\to i+1}^{\left(s\right)}\left(k|k\right) & =x\left(k\right)-{\hat{x}}_{1\to i+1}^{\left(s\right)}\left(k|k\right)\\ \; & ={\tilde{x}}^{\left(s\right)}\left(k|k-1\right)-K_{1\to i+1}^{\left(s\right)}\left(k\right)\;\varepsilon_{1\to i+1}^{\left(s,r\right)}{\left(k\right)}^{T}\\ \; & =\tilde{x}\left(k|k-1\right)-\sum_{j=1}^{i}K_{j}^{\left(s\right)}\left(k\right)\;\varepsilon_{j}^{\left(s,r\right)}\left(k\right)-K_{i+1}^{\left(s\right)}\left(k\right)\;\varepsilon_{i+1}^{\left(s,r\right)}\left(k\right) \end{array}$$

(98) $$\begin{array}{cc} P_{1\to i+1}^{\left(s\right)}\left(k|k\right) & =E\left\{{\tilde{x}}_{1\to i+1}^{\left(s\right)}\left(k|k\right)\;{\tilde{x}}_{1\to i+1}^{\left(s\right)}{\left(k|k\right)}^{T}\right\}\\ \; & =P\left(k|k-1\right)-K_{1\to i+1}^{\left(s\right)}\left(k\right)\;Q_{\varepsilon_{1\to i+1}^{\left(s,r\right)}}\left(k\right)\;K_{1\to i+1}^{\left(s\right)}{\left(k\right)}^{T}\\ \; & =P\left(k|k-1\right)-K_{1\to i}^{\left(s\right)}\left(k\right)\;Q_{\varepsilon_{1\to i}^{\left(s,r\right)}}\left(k\right)\;K_{1\to i+1}^{\left(s\right)}{\left(k\right)}^{T}-K_{i+1}^{\left(s\right)}\left(k\right)\;Q_{\varepsilon_{i+1}^{\left(s,r\right)}}\left(k\right)\;K_{i+1}^{\left(s\right)}{\left(k\right)}^{T}\\ \; & =P\left(k|k-1\right)-\sum_{j=1}^{i}K_{j}^{\left(s\right)}\left(k\right)\;Q_{\varepsilon_{j}^{\left(s,r\right)}}\left(k\right)\;K_{j}^{\left(s\right)}{\left(k\right)}^{T}-K_{i+1}^{\left(s\right)}\left(k\right)\;Q_{\varepsilon_{i+1}^{\left(s,r\right)}}\left(k\right)\;K_{i+1}^{\left(s\right)}{\left(k\right)}^{T} \end{array}$$

(99) $$K_{i+1}^{\left(s\right)}\left(k\right)=P_{{\tilde{x}}_{i}\;\varepsilon_{i+1}^{\left(s,r\right)}}\left(k|k-1\right)\;Q_{\varepsilon_{i+1}^{\left(s,r\right)}}{\left(k\right)}^{-1}$$

where

$$\begin{array}{c} Q_{\varepsilon_{i+1}^{\left(s,r\right)}}\left(k\right)=E\left\{\varepsilon_{i+1}^{\left(s,r\right)}\left(k\right)\varepsilon_{i+1}^{\left(s,r\right)}{\left(k\right)}^{T}\right\}\\ =E\left\{{\bar{\varepsilon }}_{i+1}^{\left(s,r\right)}\left(k\right){\bar{\varepsilon }}_{i+1}^{\left(s,r\right)}{\left(k\right)}^{T}\right\}-\sum_{j=1}^{i+1}Q_{{\bar{\varepsilon }}_{i+1}^{\left(s,r\right)}\varepsilon_{j}^{\left(s,r\right)}}\left(k\right)M_{i+1|j}^{T}\left(k\right)\\ =H_{i+1}\left(k\right)P_{i}^{\left(s\right)}(k\left|k\right)H_{i+1}^{T}\left(k\right)+H_{i+1}\left(k\right)P_{{\tilde{x}}_{i}v_{i+1}}\left(k\right)+P_{{\tilde{x}}_{i}v_{i+1}}^{T}\left(k\right)H_{i+1}^{T}\left(k\right)\\ +R_{i+1}\left(k\right)-\sum_{j=1}^{i+1}Q_{{\bar{\varepsilon }}_{i+1}^{\left(s,r\right)}\varepsilon_{j}^{\left(s,r\right)}}\left(k\right)M_{i+1|j}^{T}\left(k\right) \end{array}$$

$$\begin{array}{c} P_{{\tilde{x}}_{i}\varepsilon_{i+1}^{\left(s,r\right)}}\left(k|k-1\right)=E\left\{{\tilde{x}}_{i}^{\left(s\right)}(k|k)\varepsilon_{i+1}^{\left(s,r\right)}{\left(k\right)}^{T}\right\}\\ =E\{{\tilde{x}}_{i}^{\left(s\right)}(k|k){\left[H_{i+1}\left(k\right){\tilde{x}}_{i}^{\left(s\right)}(k|k)+v_{i+1}\left(k\right)\right]}^{T}\}\\ =P_{i}^{\left(s\right)}\left(k|k\right)H_{i+1}^{T}\left(k\right)+P_{{\tilde{x}}_{i}v_{i+1}}\left(k\right) \end{array}$$

(3) An equivalence relation is established between the innovation of the centralized filter and the sequential innovation, due to

$$\begin{array}{c} \;\varepsilon_{i+1}^{\left(s,r\right)}\left(k\right)=\;{\bar{\varepsilon }}_{i+1}^{\left(s,r\right)}\left(k\right)-\sum_{j=1}^{i}M_{i+1|j}\left(k\right)\varepsilon_{j}^{\left(s,r\right)}\left(k\right)\\ \;\;\;\;\;\;\;\;\;\;\;\;=H_{i+1}\left(k\right){\tilde{x}}_{i}^{\left(s\right)}(k|k)+v_{i+1}\left(k\right)-\sum_{j=1}^{i}M_{i+1|j}\left(k\right)\varepsilon_{j}^{\left(s,r\right)}\left(k\right)\\ =H_{i+1}\left(k\right)\left[\tilde{x}(k|k-1)-\sum_{j=1}^{i}K_{j}^{\left(s\right)}\left(k\right)\varepsilon_{j}^{\left(s,r\right)}(k)\right]+v_{i+1}\left(k\right)-\sum_{j=1}^{i}M_{i+1|j}\left(k\right)\varepsilon_{j}^{\left(s,r\right)}\left(k\right)\\ =H_{i+1}\left(k\right)\tilde{x}(k|k-1)+v_{i+1}\left(k\right)-\sum_{j=1}^{i}\left[H_{i+1}\left(k\right)K_{j}^{\left(s\right)}\left(k\right)+M_{i+1|j}\left(k\right)\right]\varepsilon_{j}^{\left(s,r\right)}(k)\\ =\varepsilon_{i+1}^{\left(c,r\right)}\left(k\right)-\sum_{j=1}^{i}{\bar{M}}_{i+1|j}\left(k\right)\varepsilon_{j}^{\left(s,r\right)}(k) \end{array}$$

(100) $$\varepsilon_{i+1}^{\left(c,r\right)}\left(k\right)=\sum_{j=1}^{i}{\bar{M}}_{i+1|j}\left(k\right)\;\varepsilon_{j}^{\left(s,r\right)}\left(k\right)+\varepsilon_{i+1}^{\left(s,r\right)}\left(k\right)$$

Therefore, we have

(101) $${\bar{M}}_{i+1|j}\left(k\right)=H_{i+1}\left(k\right)\;K_{j}^{\left(s\right)}\left(k\right)+M_{i+1|j}\left(k\right)$$

(102) $$M_{i+1,i+1}^{\left(c,s\right)}\left(k\right)=\left[\begin{array}{cc} M_{i,i}^{\left(c,s\right)}\left(k\right) & 0\\ {\hat{M}}_{i+1}^{\left(c,s\right)}\left(k\right) & I_{i+1} \end{array}\right]=\left[\begin{array}{ccccc} I_{1} & 0 & \cdots & 0 & 0\\ {\bar{M}}_{2|1} & I_{2} & \cdots & 0 & 0\\ \vdots & \vdots & \ddots & \vdots & \vdots \\ {\bar{M}}_{i|1} & {\bar{M}}_{i|2} & \cdots & I_{i} & 0\\ {\bar{M}}_{i+1|1} & {\bar{M}}_{i+1|2} & \cdots & {\bar{M}}_{i+1|i} & I_{i+1} \end{array}\right],\;\left|M_{i+1,i+1}^{\left(c,s\right)}\left(k\right)\right|\neq 0$$

$$\varepsilon_{1\to i+1}^{\left(c,f\right)}\left(k\right)=M_{i+1,i+1}^{\left(c,s\right)}\left(k\right)\varepsilon_{1\to i+1}^{\left(s,f\right)}\left(k\right)=\left[\begin{array}{cc} M_{i,i}^{\left(c,s\right)}\left(k\right) & 0\\ {\hat{M}}_{i+1}^{\left(c,s\right)}\left(k\right) & I_{i+1} \end{array}\right]\left[\begin{array}{c} \varepsilon_{1\to i}^{\left(s,f\right)}\left(k\right)\\ \varepsilon_{i+1}^{\left(s,f\right)}\left(k\right) \end{array}\right]$$

$$\varepsilon_{1\to i+1}^{\left(c,r\right)}\left(k\right)=M_{i+1,i+1}^{\left(c,s\right)}\left(k\right)\varepsilon_{1\to i+1}^{\left(s,r\right)}\left(k\right)=\left[\begin{array}{cc} M_{i,i}^{\left(c,s\right)}\left(k\right) & 0\\ {\hat{M}}_{i+1}^{\left(c,s\right)}\left(k\right) & I_{i+1} \end{array}\right]\left[\begin{array}{c} \varepsilon_{1\to i}^{\left(s,r\right)}\left(k\right)\\ \varepsilon_{i+1}^{\left(s,r\right)}\left(k\right) \end{array}\right]$$

(4) Equivalence analysis between the innovation gain of the centralized filter and the sequential innovation gain:

(103) $$K_{1\to i+1}^{\left(c\right)}\left(k\right)\;\varepsilon_{1\to i+1}^{\left(c,f\right)}\left(k\right)=\sum_{j=1}^{i}K_{j}^{\left(s\right)}\left(k\right)\;\varepsilon_{j}^{\left(s,f\right)}\left(k\right)+K_{i+1}^{\left(s\right)}\left(k\right)\;\varepsilon_{i+1}^{\left(s,f\right)}\left(k\right)$$

$$\begin{array}{c} K_{1\to i+1}^{\left(c\right)}(k)\varepsilon_{1\to i+1}^{\left(c,f\right)}(k)\\ =E\left\{\tilde{x}\left(k|k-1\right)\varepsilon_{1\to i+1}^{\left(c,r\right)}(k{)}^{T}\right\}E{\left\{\varepsilon_{1\to i+1}^{\left(c,r\right)}(k)\varepsilon_{1\to i+1}^{\left(c,r\right)}(k{)}^{T}\right\}}^{-1}\varepsilon_{1\to i+1}^{\left(c,f\right)}(k)\\ =E\left\{\tilde{x}\left(k|k-1\right){\left[\begin{array}{c} \varepsilon_{1\to i}^{\left(s,r\right)}(k)\\ \varepsilon_{i+1}^{\left(s,r\right)}(k) \end{array}\right]}^{T}\right\}M_{i+1,i+1}^{\left(c,s\right)}{\left(k\right)}^{T}\\ \times {\left(M_{i+1,i+1}^{\left(c,s\right)}{\left(k\right)}^{T}\right)}^{-1}E{\left\{\left[\begin{array}{c} \varepsilon_{1\to i}^{\left(s,r\right)}(k)\\ \varepsilon_{i+1}^{\left(s,r\right)}(k) \end{array}\right]{\left[\begin{array}{c} \varepsilon_{1\to i}^{\left(s,r\right)}(k)\\ \varepsilon_{i+1}^{\left(s,r\right)}(k) \end{array}\right]}^{T}\right\}}^{-1}M_{i+1,i+1}^{\left(c,s\right)}{\left(k\right)}^{-1}M_{i+1,i+1}^{\left(c,s\right)}\left(k\right)\left[\begin{array}{c} \varepsilon_{1\to i}^{\left(s,f\right)}(k)\\ \varepsilon_{i+1}^{\left(s,f\right)}(k) \end{array}\right]\\ =\left[E\left\{\tilde{x}\left(k|k-1\right)\varepsilon_{1\to i}^{\left(s,r\right)}{\left(k\right)}^{T}\right\},E\left\{\tilde{x}\left(k|k-1\right)\varepsilon_{i+1}^{\left(s,r\right)}{\left(k\right)}^{T}\right\}\right]\left[\begin{array}{cc} Q_{\varepsilon_{1\to i}^{\left(s,r\right)}}{\left(k\right)}^{-1} & 0\\ 0 & Q_{\varepsilon_{i+1}^{\left(s,r\right)}}{\left(k\right)}^{-1} \end{array}\right]\left[\begin{array}{c} \varepsilon_{1\to i}^{\left(s,f\right)}(k)\\ \varepsilon_{i+1}^{\left(s,f\right)}(k) \end{array}\right]\\ =\left[P_{\tilde{x}\varepsilon_{1\to i}^{\left(s,r\right)}}\left(k\right),E\left\{{\tilde{x}}_{i}\left(k|k\right)\varepsilon_{i+1}^{\left(s,r\right)}{\left(k\right)}^{T}\right\}\right]\left[\begin{array}{cc} Q_{\varepsilon_{1\to i}^{\left(s,r\right)}}{\left(k\right)}^{-1} & 0\\ 0 & Q_{\varepsilon_{i+1}^{\left(s,r\right)}}{\left(k\right)}^{-1} \end{array}\right]\left[\begin{array}{c} \varepsilon_{1\to i}^{\left(s,f\right)}(k)\\ \varepsilon_{i+1}^{\left(s,f\right)}(k) \end{array}\right]\\ =\left[P_{\tilde{x}\varepsilon_{1\to i}^{\left(s,r\right)}}\left(k\right),P_{{\tilde{x}}_{i}\varepsilon_{i+1}^{\left(s,r\right)}}\left(k\right)\right]\left[\begin{array}{cc} Q_{\varepsilon_{1\to i}^{\left(s,r\right)}}{\left(k\right)}^{-1} & 0\\ 0 & Q_{\varepsilon_{i+1}^{\left(s,r\right)}}{\left(k\right)}^{-1} \end{array}\right]\left[\begin{array}{c} \varepsilon_{1\to i}^{\left(s,f\right)}(k)\\ \varepsilon_{i+1}^{\left(s,f\right)}(k) \end{array}\right]\\ =P_{\tilde{x}\varepsilon_{1\to i}^{\left(s,r\right)}}\left(k\right)Q_{\varepsilon_{1\to i}^{\left(s,r\right)}}{\left(k\right)}^{-1}\varepsilon_{1\to i}^{\left(s,f\right)}(k)+P_{{\tilde{x}}_{i}\varepsilon_{i+1}^{\left(s,r\right)}}\left(k\right)Q_{\varepsilon_{i+1}^{\left(s,r\right)}}{\left(k\right)}^{-1}\varepsilon_{i+1}^{\left(s,f\right)}(k)\\ =\sum_{j=1}^{i}K_{j}^{\left(s\right)}\left(k\right)\varepsilon_{j}^{\left(s,f\right)}(k)+K_{i+1}^{\left(s\right)}\left(k\right)\varepsilon_{i+1}^{\left(s,f\right)}(k) \end{array}$$

(5) Equality of the estimation error covariance matrix between the centralized filter and the sequential filter:

(104) $$K_{1\to i+1}^{\left(c\right)}\left(k\right)\;Q_{\varepsilon_{1\to i+1}^{\left(c,r\right)}}\left(k\right)\;K_{1\to i+1}^{\left(c\right)}{\left(k\right)}^{T}=\sum_{j=1}^{i}K_{j}^{\left(s\right)}\left(k\right)\;Q_{\varepsilon_{j}^{\left(s,r\right)}}\left(k\right)\;K_{j}^{\left(s\right)}{\left(k\right)}^{T}+K_{i+1}^{\left(s\right)}\left(k\right)\;Q_{\varepsilon_{i+1}^{\left(s,r\right)}}\left(k\right)\;K_{i+1}^{\left(s\right)}{\left(k\right)}^{T}$$

$$\begin{array}{c} K_{1\to i+1}^{\left(c\right)}(k)Q_{\varepsilon_{1\to i+1}^{\left(c,r\right)}}(k)K_{1\to i+1}^{\left(c\right)}(k{)}^{T}\\ =E\left\{\tilde{x}\left(k|k-1\right)\varepsilon_{1\to i+1}^{\left(c,r\right)}{\left(k\right)}^{T}\right\}E{\left\{\varepsilon_{1\to i+1}^{\left(c,r\right)}\left(k\right)\varepsilon_{1\to i+1}^{\left(c,r\right)}{\left(k\right)}^{T}\right\}}^{-1}E\left\{\varepsilon_{1\to i+1}^{\left(c,r\right)}\left(k\right)\varepsilon_{1\to i+1}^{\left(c,r\right)}{\left(k\right)}^{T}\right\}\\ \times E{\left\{\varepsilon_{1\to i+1}^{\left(c,r\right)}\left(k\right)\varepsilon_{1\to i+1}^{\left(c,r\right)}{\left(k\right)}^{T}\right\}}^{-1}E{\left\{\tilde{x}\left(k|k-1\right)\varepsilon_{1\to i+1}^{\left(c,r\right)}{\left(k\right)}^{T}\right\}}^{T}\\ =E\left\{\tilde{x}\left(k|k-1\right)\varepsilon_{1\to i+1}^{\left(c,r\right)}{\left(k\right)}^{T}\right\}E{\left\{\varepsilon_{1\to i+1}^{\left(c,r\right)}\left(k\right)\varepsilon_{1\to i+1}^{\left(c,r\right)}{\left(k\right)}^{T}\right\}}^{-1}E{\left\{\tilde{x}\left(k|k-1\right)\varepsilon_{1\to i+1}^{\left(c,r\right)}{\left(k\right)}^{T}\right\}}^{T}\\ =E\left\{\tilde{x}\left(k|k-1\right){\left[\begin{array}{c} \varepsilon_{1\to i}^{\left(s,r\right)}(k)\\ \varepsilon_{i+1}^{\left(s,r\right)}(k) \end{array}\right]}^{T}\right\}M_{i+1,i+1}^{\left(c,s\right)}{\left(k\right)}^{T}{\left(M_{i+1,i+1}^{\left(c,s\right)}{\left(k\right)}^{T}\right)}^{-1}E\left\{\left[\begin{array}{c} \varepsilon_{1\to i}^{\left(s,r\right)}(k)\\ \varepsilon_{i+1}^{\left(s,r\right)}(k) \end{array}\right]{\left[\begin{array}{c} \varepsilon_{1\to i}^{\left(s,r\right)}(k)\\ \varepsilon_{i+1}^{\left(s,r\right)}(k) \end{array}\right]}^{T}\right\}\\ \times M_{i+1,i+1}^{\left(c,s\right)}{\left(k\right)}^{-1}M_{i+1,i+1}^{\left(c,s\right)}\left(k\right)E{\left\{\tilde{x}\left(k|k-1\right){\left[\begin{array}{c} \varepsilon_{1\to i}^{\left(s,r\right)}(k)\\ \varepsilon_{i+1}^{\left(s,r\right)}(k) \end{array}\right]}^{T}\right\}}^{T}\\ =\left[P_{\tilde{x}\varepsilon_{1\to i}^{\left(s,r\right)}}\left(k\right),P_{{\tilde{x}}_{i}\varepsilon_{i+1}^{\left(s,r\right)}}\left(k\right)\right]\left[\begin{array}{cc} Q_{\varepsilon_{1\to i}^{\left(s,r\right)}}{\left(k\right)}^{-1} & 0\\ 0 & Q_{\varepsilon_{i+1}^{\left(s,r\right)}}{\left(k\right)}^{-1} \end{array}\right]{\left[P_{\tilde{x}\varepsilon_{1\to i}^{\left(s,r\right)}}\left(k\right),P_{{\tilde{x}}_{i}\varepsilon_{i+1}^{\left(s,r\right)}}\left(k\right)\right]}^{T}\\ =P_{\tilde{x}\varepsilon_{1\to i}^{\left(s,r\right)}}\left(k\right)Q_{\varepsilon_{1\to i}^{\left(s,r\right)}}{\left(k\right)}^{-1}P_{\tilde{x}\varepsilon_{1\to i}^{\left(s,r\right)}}{\left(k\right)}^{T}+P_{{\tilde{x}}_{i}\varepsilon_{i+1}^{\left(s,r\right)}}\left(k\right)Q_{\varepsilon_{i+1}^{\left(s,r\right)}}{\left(k\right)}^{-1}P_{{\tilde{x}}_{i}\varepsilon_{i+1}^{\left(s,r\right)}}{\left(k\right)}^{T}\\ =\sum_{j=1}^{i}K_{j}^{\left(s\right)}\left(k\right)Q_{\varepsilon_{j}^{\left(s,r\right)}}\left(k\right)K_{j}^{\left(s\right)}{\left(k\right)}^{T}+K_{i+1}^{\left(s\right)}\left(k\right)Q_{\varepsilon_{i+1}^{\left(s,r\right)}}\left(k\right)K_{i+1}^{\left(s\right)}{\left(k\right)}^{T} \end{array}$$

### 5.4. The Centralized Fusion Kalman Filter Composed of Sensors and the Corresponding Sequential Fusion Kalman Filter Are Obtained

(1) These are formally equivalent:

(105) $${\hat{x}}_{1\to N}^{\left(c\right)}\left(k|k\right)={\hat{x}}_{1\to N}^{\left(s\right)}\left(k|k\right)$$

where

(106) $$\begin{array}{cc} {\hat{x}}_{1\to N}^{\left(c\right)}\left(k|k\right) & =E\{x\left(k\right)|\hat{x}\left(k-1|k-1\right),y_{1}\left(k\right),y_{2}\left(k\right),\ldots ,y_{i}\left(k\right),\ldots ,y_{N}\left(k\right)\}\\ \; & =\hat{x}\left(k|k-1\right)+K_{1\to N}^{\left(c\right)}\left(k\right)\;\varepsilon_{1\to N}^{\left(c,f\right)}\left(k\right) \end{array}$$

(107) $$\begin{array}{cc} {\hat{x}}_{1\to N}^{\left(s\right)}\left(k|k\right) & =E\{x\left(k\right)|\hat{x}\left(k-1|k-1\right),y_{1}\left(k\right),y_{2}\left(k\right),\ldots ,y_{i}\left(k\right),\ldots ,y_{N}\left(k\right)\}\\ \; & =\hat{x}\left(k|k-1\right)+\sum_{i=1}^{N}K_{i}^{\left(c\right)}\left(k\right)\;\varepsilon_{i}^{\left(c,f\right)}\left(k\right) \end{array}$$

(108) $$K_{1\to N}^{\left(c\right)}\left(k\right)\;\varepsilon_{1\to N}^{\left(c,f\right)}\left(k\right)=\sum_{i=1}^{N}K_{i}^{\left(c\right)}\left(k\right)\;\varepsilon_{i}^{\left(c,f\right)}\left(k\right)$$

(2) These are equivalent in performance:

(109) $$P_{1\to N}^{\left(c\right)}\left(k|k\right)=P_{1\to N}^{\left(s\right)}\left(k|k\right)$$

(110) $$P_{1\to N}^{\left(c\right)}\left(k|k\right)=P\left(k|k-1\right)-K_{1\to N}^{\left(c\right)}\left(k\right)\;Q_{\varepsilon_{1\to N}^{\left(c,r\right)}}\left(k\right)\;K_{1\to N}^{\left(c\right)}{\left(k\right)}^{T}$$

(111) $$P_{1\to N}^{\left(s\right)}\left(k|k\right)=P\left(k|k-1\right)-\sum_{i=1}^{N}K_{i}^{\left(s\right)}\left(k\right)\;Q_{\varepsilon_{i}^{\left(s,r\right)}}\left(k\right)\;K_{i}^{\left(s\right)}{\left(k\right)}^{T}$$

(112) $$K_{1\to N}^{\left(c\right)}\left(k\right)\;Q_{\varepsilon_{1\to N}^{\left(c,r\right)}}\left(k\right)\;K_{1\to N}^{\left(c\right)}{\left(k\right)}^{T}=\sum_{i=1}^{N}K_{i}^{\left(s\right)}\left(k\right)\;Q_{\varepsilon_{i}^{\left(s,r\right)}}\left(k\right)\;K_{i}^{\left(s\right)}{\left(k\right)}^{T}$$

## 6. Simulation Research

Consider a uniform linear motion system, where the measurement system consists of three sensors.

$$x\left(k+1\right)=\left[\begin{array}{ccc} 1 & T & T^{2}/2\\ 0 & 1 & T\\ 0 & 0 & 1 \end{array}\right]x\left(k\right)+\Gamma \left(k\right)\xi \left(k\right)$$

$$y_{i}\left(k\right)=H_{i}x_{i}\left(k\right)+v_{i}\left(k\right),i=1,2,3$$

$$v_{i}\left(k\right)=\beta_{i}\xi \left(k-1\right)+\eta_{i}\left(k\right),i=1,2,3$$

Let the sampling time be T=0.2 and $x\left(k\right)={\left[\begin{array}{ccc} S(k) & \dot{S}\left(k\right) & \ddot{S}\left(k\right) \end{array}\right]}^{T}$, where S(k), $\dot{S}\left(k\right)$ and $\ddot{S}\left(k\right)$ denote the position, velocity and acceleration of the target, respectively. ξ(k)∈R is the system noise, which is a zero-mean Gaussian white noise with variance $\sigma_{\xi }^{2}$, and $\Gamma \left(k\right)={\left[\begin{array}{ccc} T^{2}/2 & T & 1 \end{array}\right]}^{T}$ is the noise transition matrix. $\eta_{i}\left(k\right),i=1,2,3$ are zero-mean Gaussian white noises with variance $\sigma_{\eta_{i}}^{2}$, and ξ(k) is mutually independent of $\eta_{i}\left(k\right),i=1,2,3$. $y_{i}\left(k\right),i=1,2,3$ are the measurements from three sensors, which observe the position, velocity and acceleration, respectively, i.e., $H_{1}\left(k\right)=\left[\begin{array}{ccc} 1 & 0 & 0 \end{array}\right]$, $H_{2}\left(k\right)=\left[\begin{array}{ccc} 0 & 1 & 0 \end{array}\right]$ and $H_{3}\left(k\right)=\left[\begin{array}{ccc} 0 & 0 & 1 \end{array}\right]$.

Let the system noise be w(k)=Γ(k)ξ(k). Then, the system noise covariance is $Q\left(k\right)=\Gamma \left(k\right)\Gamma^{T}\left(k\right)\sigma_{\xi }^{2}$. The measurement noises $v_{i}\left(k\right),i=1,2,3$ are zero-mean with covariance $R_{i,i}\left(k\right)=\beta_{i}\sigma_{\xi }^{2}+\sigma_{\eta_{i}}^{2}$ and for i≠j, $R_{i,j}\left(k\right)=\beta_{i}\beta_{j}\sigma_{\xi }^{2}$. In addition, the cross-covariance between the measurement noise $v_{i}\left(k\right)$ and the system noise $w_{i}\left(k\right)$ is $S_{i}\left(k\right)=\beta_{i}\sigma_{\xi }^{2}\Gamma \left(k-1\right)$, where the degree of correlation is determined by the magnitude of $\beta_{i}$.

In the simulation experiments, we set $\beta_{1}=3$, $\beta_{2}=2$, $\beta_{3}=1$, $\sigma_{\xi }^{2}=0.01$, $\sigma_{\eta_{1}}^{2}=0.16$, $\sigma_{\eta_{2}}^{2}=0.09$ and $\sigma_{\eta_{3}}^{2}=0.04$, with the initial values $x_{0}=0$ and $P_{0}=I_{3}$.

**Figure 1:**
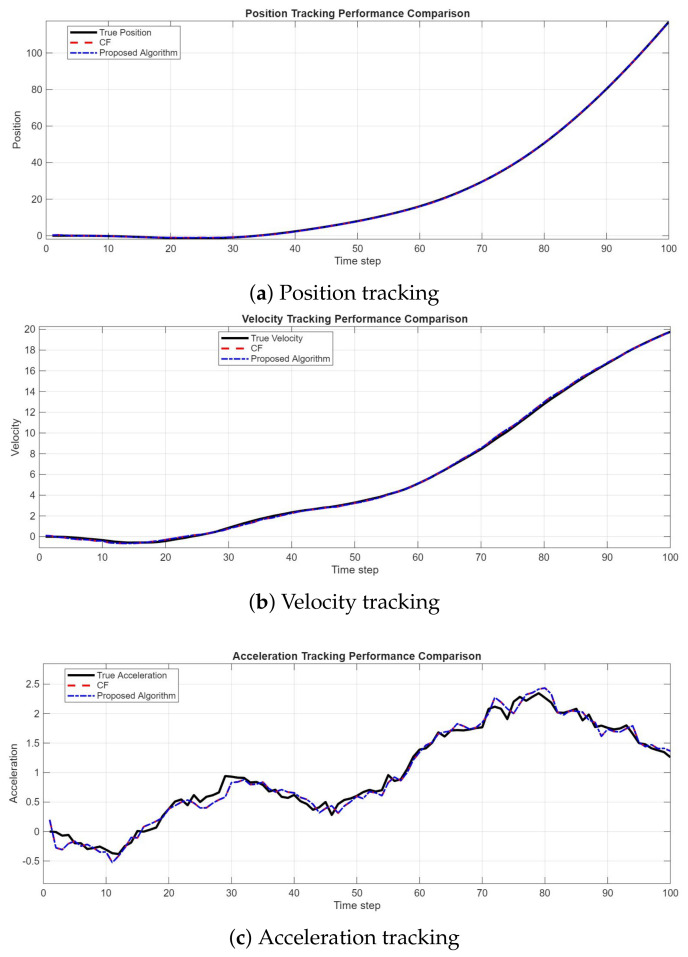
Tracking performance comparison between Proposed Algorithm and CF.

**Figure 2:**
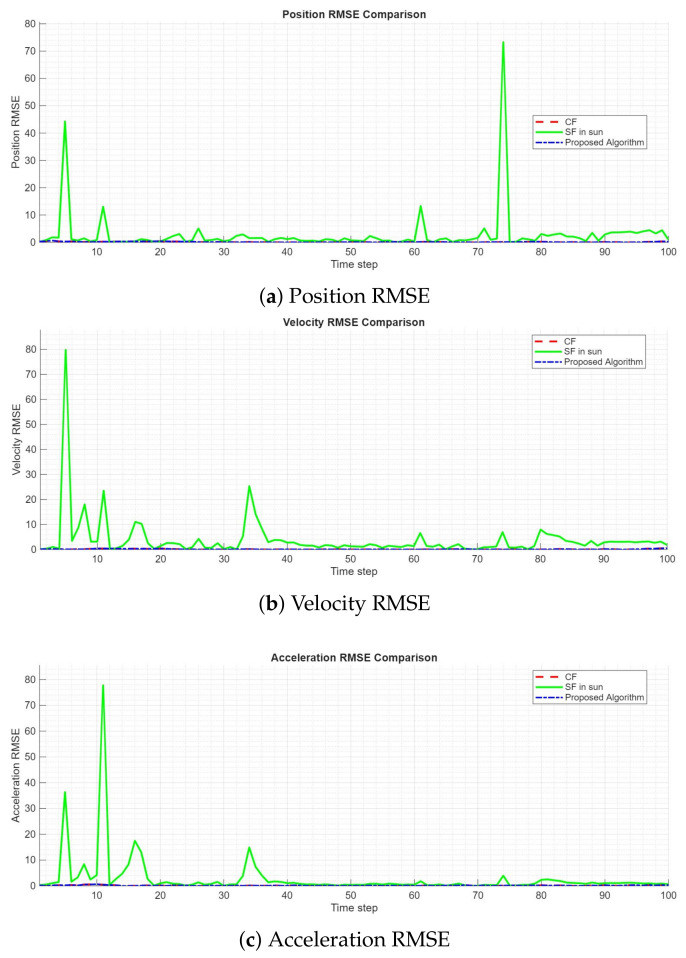
Comparison of RMSE between the proposed algorithm and SF in Sun [26].

We take 100 time sampling points. Table 1 and Table 2 and Figure 1 and Figure 2 show the results of 100 Monte Carlo simulations.

Figure 1 presents the tracking performance comparison curves for position, velocity, and acceleration, respectively. Over all time steps, the estimation curves of the proposed algorithm and the centralized algorithm completely overlap with each other and closely follow the true trajectory, intuitively verifying the equivalence of the two algorithms—indicating not only consistent statistical accuracy but also identical instantaneous estimation performance. At the final time step, the estimated trajectories of all three states tend to stabilize, with no cumulative amplification of deviations from the true trajectories, demonstrating that both algorithms possess good steady-state convergence and meet the stability requirements of linear system filtering.

Table 1 presents the true values and state estimation results at the final time. From the results in Table 1, it can be concluded that, under the same initial conditions, the centralized fusion Kalman filter algorithm for dual-correlated noises and the sequential fusion Kalman filter algorithm established in this paper yield identical estimation results, which verifies the conclusion of Theorem 1.

To further demonstrate the advantages of our proposed algorithm, under the same initial conditions, a comparison is made with the sequential algorithm of Sun [26] for the case of two sensors. The simulation results are shown in Figure 2 as the RMSE comparison plot. It can be seen from Figure 2 that the proposed algorithm achieves the same estimation accuracy as the centralized fusion Kalman filter and outperforms the sequential algorithm of Sun [26] in terms of accuracy. This is because the definition of the sequential innovation in Sun [26] is flawed, leading to the invalidity of the claim that their sequential algorithm possesses global optimality. Moreover, Table 2 presents the average RMSE corresponding to Figure 2, and the numerical results lead to the same conclusion.

**Remark 2.**  *The deviation between the estimated and true acceleration in Table 1 arises from the transient filter response caused by the initial conditions and noise correlation, which does not fully decay within the finite simulation steps. In 100 Monte Carlo runs, the mean estimation error is approximately 0.1044 and the standard deviation is approximately 0.085; the deviation is less than twice the standard deviation, indicating that it falls within the normal range of statistical fluctuation. The two algorithms produce exactly the same outputs, confirming that this deviation is not an inherent bias of the algorithm and does not affect their equivalence. Therefore, this deviation is statistically acceptable.*

## 7. Conclusions

For multi-sensor systems with correlated noises, where the process noise is correlated with the measurement noise of different sensors and the measurement noises across different sensors are mutually correlated, a mutually orthogonal sequential innovation sequence is constructed based on the Gram–Schmidt orthogonalization principle. Accordingly, a sequential fusion filter is proposed that is performance-equivalent to the centralized fusion filter. Compared with the centralized fusion filter, the proposed sequential fusion algorithm avoids the augmentation of measurement equations, thereby reducing the computational cost while maintaining identical estimation accuracy. Rigorous theoretical proof shows that the proposed sequential fusion filter and the centralized fusion filter achieve exactly the same estimation performance. Meanwhile, the sequential fusion filter is recursive in nature, which makes it suitable for real-time applications.

## Figures and Tables

**Table 1:** True values and estimated values of the CF and the Proposed Algorithm.

State	True Value	CF	Proposed Algorithm
Position	116.9901	116.8565	116.8565
Velocity	19.7681	19.7632	19.7632
Acceleration	1.2607	1.3651	1.3651

**Table 2:** Comparison of average RMSE among the SF in Sun [26], CF, and the Proposed Algorithm.

Fusion Algorithms	Position RMSE	Velocity RMSE	Acceleration RMSE
SF in Sun [26]	2.527351	2.997220	2.478368
CF	0.140884	0.122696	0.124892
Proposed Algorithm	0.140884	0.122696	0.124892

## Data Availability

The original contributions presented in this study are included in the article. Further inquiries can be directed to the corresponding author(s).

## References

[1] Sun S., Lin H., Ma J., Li X. (2017). Multi-sensor distributed fusion estimation with applications in networked systems: A review paper. Inf. Fusion.

[2] Elkhalea M.F., Hendy H., Kamel A., Abosekeen A., Noureldin A. (2025). Centralized measurement level fusion of GNSS and inertial sensors for robust positioning and navigation. Sensors.

[3] Qiu S., Zhao H., Jiang N., Wang Z., Liu L., An Y., Zhao H., Miao X., Liu R., Fortino G. (2022). Multi-sensor information fusion based on machine learning for real applications in human activity recognition: State-of-the-art and research challenges. Inf. Fusion.

[4] Fu J., Sun G., Yao W., Wu L. (2022). On trajectory homotopy to explore and penetrate dynamically of multi-UAV. IEEE Trans. Intell. Transp. Syst..

[5] Sharma D., Kumar A., Tyagi N., Chavan S.S., Gangadharan S.M.P. (2024). Towards intelligent industrial systems: A comprehensive survey of sensor fusion techniques in IIoT. Meas. Sens..

[6] He X., Hu C., Hong Y., Shi L., Fang H.T. (2020). Distributed Kalman filters with state equality constraints: Time-based and event-triggered communications. IEEE Trans. Autom. Control.

[7] Hu Z., Chen B., Zhang W.A., Yu L. (2023). Event-triggered fusion estimation under bounded noises. Automatica.

[8] Zhang R., Lin H. (2024). Event-triggered distributed fusion estimator for asynchronous Markov jump systems with correlated noises and fading measurements. Sensors.

[9] Xue W., Chen P., Li C., Cao Z., Zhang S. (2026). Communication-efficient asynchronous fusion for multi-radar systems via state and covariance projection. Electronics.

[10] Deng Z.L., Gao Y., Mao L., Li Y., Hao G. (2005). New approach to information fusion steady-state Kalman filtering. Automatica.

[11] Sun S.L. (2004). Multi-sensor information fusion white noise filter weighted by scalars based on Kalman predictor. Automatica.

[12] Geng H., Wang Z., Chen Y., Yi X., Cheng Y. (2022). Variance-constrained filtering fusion for nonlinear cyber–physical systems with the denial-of-service attacks and stochastic communication protocol. IEEE/CAA J. Autom. Sin..

[13] Lu S., Liu H. (2026). Event-triggered robust fusion estimation for multi-sensor systems under random packet drops. Signals.

[14] Shan X., Cabani A., Chafouk H. (2024). Interval split covariance intersection filter: Theory and its application to cooperative localization in a multi-sensor multi-vehicle system. Sensors.

[15] Ran C., Gao Y., Deng Z. (2024). Robust sequential fusion Kalman estimators with asymptotic equivalence and stability for networked uncertain sensor systems. Commun. Nonlinear Sci. Numer. Simul..

[16] Anderson B.D.O., Moore J.B. (1979). Optimal Filtering.

[17] Zhang W.A., Shi L. (2018). Sequential fusion estimation for clustered sensor networks. Automatica.

[18] Gao X., Chen J., Tao D., Li X. (2011). Multi-sensor centralized fusion without measurement noise covariance by variational Bayesian approximation. IEEE Trans. Aerosp. Electron. Syst..

[19] Zhu H., Luo M. (2020). Hybrid robust sequential fusion estimation for WSN-assisted moving-target localization with sensor-node-position uncertainty. IEEE Trans. Instrum. Meas..

[20] Zhao X., Yardim C., Wang D., Howe B.M. (2017). Estimating range-dependent evaporation duct height. J. Atmos. Ocean. Technol..

[21] Zeng G., Chen S., Wu H., Yang M. (2024). Continuous-discrete extended Kalman filtering based on the neural ordinary differential equations method. Eng. Appl. Artif. Intell..

[22] Wen C., Wen C., Li Y. (2008). An optimal sequential decentralized filter of discrete-time systems with cross-correlated noises. IFAC Proc. Vol..

[23] Feng X.L., Ge Q.B., Wen C.L. An optimal sequential filter for the linear system with correlated noises. Proceedings of the 2009 Chinese Control and Decision Conference.

[24] Feng X.L., Wen C.L., Xu L.Z. A new optimal OOSM filtering algorithm. Proceedings of the 2011 International Conference on Wavelet Analysis and Pattern Recognition.

[25] Yan L., Li X.R., Xia Y., Fu M. (2013). Optimal sequential and distributed fusion for state estimation in cross-correlated noise. Automatica.

[26] Lin H., Sun S. (2019). Globally optimal sequential and distributed fusion state estimation for multi-sensor systems with cross-correlated noises. Automatica.

[27] Wen C., Cai Y., Wen C., Xu X. (2013). Optimal sequential Kalman filtering with cross-correlated measurement noises. Aerosp. Sci. Technol..

[28] Huang W., Wen C. (2025). Optimal sequential fusion Kalman filter for multi-sensor linear systems with noise cross-correlated. Sensors.

